# Comparative secretomic and proteomic analysis reveal multiple defensive strategies developed by *Vibrio cholerae* against the heavy metal (Cd^2+^, Ni^2+^, Pb^2+^, and Zn^2+^) stresses

**DOI:** 10.3389/fmicb.2023.1294177

**Published:** 2023-10-26

**Authors:** Beiyu Zhang, Jingjing Xu, Meng Sun, Pan Yu, Yuming Ma, Lu Xie, Lanming Chen

**Affiliations:** ^1^Key Laboratory of Quality and Safety Risk Assessment for Aquatic Products on Storage and Preservation (Shanghai), Ministry of Agriculture and Rural Affairs of the People’s Republic of China, Shanghai, China; ^2^College of Food Science and Technology, Shanghai Ocean University, Shanghai, China; ^3^Shanghai-MOST Key Laboratory of Health and Disease Genomics (Chinese National Human Genome Center at Shanghai), Institute of Genome and Bioinformatics, Shanghai Institute for Biomedical and Pharmaceutical Technologies, Shanghai, China

**Keywords:** *Vibrio cholerae*, heavy metal, secretome, proteome, tolerance mechanism, food safety

## Abstract

*Vibrio cholerae* is a common waterborne pathogen that can cause pandemic cholera in humans. The bacterium with heavy metal-tolerant phenotypes is frequently isolated from aquatic products, however, its tolerance mechanisms remain unclear. In this study, we investigated for the first time the response of such *V. cholerae* isolates (*n* = 3) toward the heavy metal (Cd^2+^, Ni^2+^, Pb^2+^, and Zn^2+^) stresses by comparative secretomic and proteomic analyses. The results showed that sublethal concentrations of the Pb^2+^ (200 μg/mL), Cd^2+^ (12.5 μg/mL), and Zn^2+^ (50 μg/mL) stresses for 2 h significantly decreased the bacterial cell membrane fluidity, but increased cell surface hydrophobicity and inner membrane permeability, whereas the Ni^2+^ (50 μg/mL) stress increased cell membrane fluidity (*p* < 0.05). The comparative secretomic and proteomic analysis revealed differentially expressed extracellular and intracellular proteins involved in common metabolic pathways in the *V. cholerae* isolates to reduce cytotoxicity of the heavy metal stresses, such as biosorption, transportation and effluxing, extracellular sequestration, and intracellular antioxidative defense. Meanwhile, different defensive strategies were also found in the *V. cholerae* isolates to cope with different heavy metal damage. Remarkably, a number of putative virulence and resistance-associated proteins were produced and/or secreted by the *V. cholerae* isolates under the heavy metal stresses, suggesting an increased health risk in the aquatic products.

## Introduction

*Vibrio cholerae* can cause pandemic cholera in humans (Baker-Austin et al., 2018). The bacterium colonizes on intestinal mucosal cells and causes watery diarrhea and vomiting, even death (Sit et al., 2022). *V. cholerae* was detected positive in a variety of aquatic products (Xu et al., 2019; Fu et al., 2020; Chen et al., 2021). Aquatic ecosystems are challenged by anthropogenic activities, such as wastes from industrial processes (e.g., tanning, electroplating, manufacturing of chemicals and textiles, mining, and smelting), agricultural fertilizers and pesticides, leading to heavy metal pollution (Zamora-Ledezma et al., 2021; Tong et al., 2022). Due to toxicity, persistence and bioaccumulation, heavy metals in aquatic environments pose a huge risk to human health, such as lead (Pb), cadmium (Cd), nickel (Ni), and zinc (Zn) (Yu S. et al., 2022). For example, Pb (II) induced persistent hypertension and myocardial dysfunction, which adversely affected the function of cardiovascular organs (Carmignani et al., 1999). Cd induced various epigenetic changes in mammalian cells, leading to the development of breast, lung, pancreas, and kidney cancers (Genchi et al., 2020). Ni exposure caused allergy, cardiovascular disease, kidney disease, pulmonary fibrosis, and lung and nasal cancers (Yang et al., 2023). Zn is one of the most crucial trace elements required for cells in animals and humans. However, consuming too much dietary Zn (>40 mg/kg) resulted in arteriosclerosis and pancreatic damage (Arslan et al., 2010). Hazardous heavy metals were detected in waters, sediments and aquatic products, particularly in developing nations (Wang et al., 2020; Zamora-Ledezma et al., 2021; Wang Y. et al., 2022). The Cd and Pb had high cytotoxicity even in low concentrations (Carmignani et al., 1999; Sadeq and Beckerman, 2019).

Low levels of heavy metals (far lower than minimal inhibitory concentrations, MICs) also enabled bacteria to obtain resistance (Li et al., 2019). Heavy metal resistant *V. cholerae* isolates have been found in aquatic products in our recent reports (Xu et al., 2019; Fu et al., 2020; Chen et al., 2021). For example, Chen et al. surveyed the prevalence of *V. cholerae* in 36 species of edible aquatic animals sampled in Fuzhou and Shanghai in 2019 in China. They found high incidence of tolerance to heavy metals Hg^2+^ (67.0%), Pb^2+^ (57.6%), and Zn^2+^ (57.6%) among *V. cholerae* isolates (*n* = 203) (Chen et al., 2021). Fu et al. investigated genetic diversity of *V. cholerae* isolates (*n* = 370) originated from 15 species of edible aquatic animals collected in 2018 in Shanghai, China. High percentages of tolerance to Hg^2+^ (69.5%), Ni^2+^ (32.4%), and Cd^2+^ (30.8%) were observed among the isolates (Fu et al., 2020). It has been reported that heavy metals in sublethal levels increased mutation rates and enriched *de novo* mutants to resist multiple antibiotics (Li et al., 2019; Zhong et al., 2021). The emergence and spread of multidrug resistant (MDR) pathogenic bacteria including *V. cholerae* has become one of the most challenging issues in clinical treatment, due to the limited therapeutic options (Das et al., 2020; Salamm et al., 2023). Therefore, to decipher molecular mechanisms underlying heavy metal tolerance of *V. cholerae* is imperative for effectively controlling infectious disease caused by the MDR pathogen.

Stress response or resistance to heavy metals are complex biological processes with numerous proteins involved or at least affected (Okay et al., 2020). Two-dimensional gel electrophoresis (2D-GE) coupled with liquid chromatography–tandem mass spectrometry (LC–MS/MS) are useful techniques for the global identification of protein changes in different organisms in response to biotic and abiotic stresses. For example, Sun et al. identified a total of 1,424 differentially expressed proteins (DEPs) in plant *Brassica campestris* L. hairy roots in response to Cd^2+^ (200 μM) stress (Sun et al., 2023). Sánchez-Rojas et al. (2022) reported 252 and 118 differentially regulated proteins in yeast *Yarrowia lipolytica* under the treatment with Cd^2+^ (0.11 mM) and Cr^6+^ (0.19 mM), respectively.

In our previous studies, the 2D-GE combined with LC–MS/MS techniques were also applied in global identification of DEPs in *Vibrio* species (Zhu et al., 2020; Shan et al., 2022; Yan et al., 2022). For example, Zhu et al. (2020) compared secretomes and proteomes of *Vibrio parahaemolyticus* strains isolated from 12 species of aquatic animals and identified 28 differential extracellular proteins. On the basis of our previous studies, in the study we deciphered molecular mechanisms underlying the heavy metal (Cd^2+^, Ni^2+^, Pb^2+^, and Zn^2+^) tolerance of *V. cholerae* isolated from aquatic animals. The major objectives of this study were: (1) to examine the growth of the *V. cholerae* isolates (*n* = 3) with heavy metal-tolerant phenotypes under different concentrations of heavy metals (3.125–3,200 μg/mL); (2) to obtain secretomes and proteomes of the *V. cholerae* isolates under the sublethal concentrations of Cd^2+^ (12.5 μg/mL), Ni^2+^(50 μg/mL), Pb^2+^ (200 μg/mL), or Zn^2+^(50 μg/mL) stresses using the 2D-GE and LC–MS/MS techniques; and (3) to figure out defensive strategies adopted by the *V. cholerae* isolates toward the Cd^2+^, Ni^2+^, Pb^2+^, and Zn^2+^ stresses. To the best of our knowledge, this study was the first to investigate the response of *V. cholerae* toward the heavy metal stresses by comparative secretomic and proteomic analyses. The results of this study facilitate the better understanding of the bacterial resistance and persistence worldwide.

## Materials and methods

### Bacterial strains and culture conditions

The non O1/O139 *V. cholerae* J9-62, Q6-10, and N9-4 strains studied in this study were isolated from edible aquatic products including fish *Carassius auratus* and *Ctenopharyngodon idellus*, and shellfish *Saxidomus purpuratus*, respectively, and their genotypes and resistance phenotypes were determined (Chen et al., 2021; Supplementary Table S1). The *V. cholerae* isolates were routinely incubated in trypsin soybean broth (TSB) (3% NaCl, pH 8.5) (Beijing Luqiao Technology Co., Ltd., Beijing, China) at 37°C with shaking at 180 rpm (Xu et al., 2019; Fu et al., 2020; Chen et al., 2021).

### Determination of MICs of heavy metals

The MICs of heavy metals against the *V. cholerae* isolates were determined using the broth dilution testing (microdilution) approved by Clinical and Laboratory Standards Institute (CLSI, M2-A9, 2006), USA. Heavy metals included CdCl_2_, NiCl_2_, PbCl_2_, and ZnCl_2_ (Sinopharm Chemical Reagent Co., Ltd., Shanghai, China). *Escherichia coli* K-12 (Institute of Industrial Microbiology, Shanghai, China) was used as a quality control strain (Xu et al., 2019; Fu et al., 2020; Chen et al., 2021).

### Growth curve assay

The *V. cholerae* isolates were incubated in the TSB medium supplemented with different concentrations (3200–3.125 μg/mL) of the heavy metals (CdCl_2_, NiCl_2_, PbCl_2_, or ZnCl_2_) at 37°C for 40 h, respectively. Growth curves were measured using Bioscreen Automatic Growth Curve Analyzer (BioTek Instruments, Inc., Winooski, VT, USA) (Yang et al., 2020). Bacterial survival was examined using the standard plate counting method (Yu P. et al., 2022). The sublethal concentrations of the heavy metals were defined as fatality rates less than 50% under the treatment conditions for 2 h (Yu P. et al., 2022).

### Scanning Electron Microscopy (SEM) analysis

The *V. cholerae* isolates were incubated in the TSB medium to the mid logarithmic growth phase (mid-LGP) at 37°C. A final concentration of Cd^2+^ (12.5 μg/mL), Ni^2+^(50 μg/mL), Pb^2+^ (200 μg/mL), or Zn^2+^(50 μg/mL) was added to the bacterial culture (5 mL), and then continuously incubated at 37°C for 2 h, 4 h, and 6 h. The cell mixture (1.5 mL) was then harvested, washed, fixed, and observed using thermal field emission SEM (Hitachi, SU5000, Tokyo, Japan, 5.0 kV, ×30,000) as described in our recent report (Yu P. et al., 2022). The untreated bacterial culture was used as a negative control.

### Bacterial cell membrane fluidity and permeability, and surface hydrophobicity assays

The *V. cholerae* isolates were treated with the heavy metals for 2 h as described in the Scanning Electron Microscopy (SEM) Analysis section. The bacterial cell membrane fluidity was examined using the 1, 6-Diphenyl-1, 3, 5-hexatrine (DPH, National Pharmaceutical Group Corporation Co., Ltd., Shanghai, China) as a probe (Voss and Montville, 2014). The bacterial inner membrane permeability was examined using the O-nitrophenyl-β-D galactopyranoside (ONPG, Beijing Solarbio Science & Technology Co., Ltd., Beijing, China) as a probe (Huang et al., 2021). The OD_415_ values were determined using BioTek Synergy 2 (BioTek, Burlington, VT, USA) every 30 min for 2 h. The bacterial cell surface hydrophobicity was measured using the n-hexadecane (National Pharmaceutical Group Corporation Co., Ltd., Shanghai, China) as a probe (Yan et al., 2016). The untreated bacterial culture was used as a negative control.

### 2D-GE analysis

The *V. cholerae* isolates were treated with the heavy metals for 2 h as described in the Scanning Electron Microscopy (SEM) Analysis section, but incubated without shaking. Extracellular proteins of the *V. cholerae* isolates were extracted as described in our recent reports (Zhu et al., 2020; Shan et al., 2022; Yan et al., 2022). Isoelectric focusing (IEF) was performed using immobilized pH gradient (IPG) gels (pH 4–7, 7 cm; Bio-Rad, Hercules, USA). The second-dimension sodium dodecyl sulfate–polyacrylamide gel electrophoresis (SDS-PAGE), gel staining, protein spot detection, spot matching, and quantitative intensity analysis were performed as described previously (Zhu et al., 2020; Shan et al., 2022; Yan et al., 2022). In addition, intracellular proteins were extracted using Bacterial Protein Extraction Kit (Shanghai Sangon Biological Engineering Technology and Service Co., Ltd., Shanghai, China), and analyzed as described previously (Zhu et al., 2020; Shan et al., 2022; Yan et al., 2022). The untreated bacterial culture was used as a negative control.

### LC–MS/MS analysis

The LC–MS/MS analysis was carried out by HooGen Biotech, Shanghai, China using Q Executive Mass Spectrometer (Thermo Fisher Scientific, Waltham, MA, USA) coupled with Easy nLC 1200 Chromatography System (Zhu et al., 2020; Shan et al., 2022; Yan et al., 2022). The automated peptide identification and protein calls were performed using Uniprot *V. cholerae* 80449 20221026 databases in Mascot version 2.2 server (Matrix Science, London, United Kingdom) with the same criteria described in our previous report (Zhu et al., 2020). A false discovery rate (FDR) was set below 0.01 for both peptides and proteins. The lable-free relative protein quantitation was performed based on peak areas of corresponding peptides. To reveal intensities that were significant between the treatment groups under the heavy metal stresses and the control group, the *p* values of <0.01, and fold changes of >1.5 were set for significant difference. Three independent biological replicates were prepared and combined for each sample.

### Quantitative reverse transcription-PCR (qRT-PCR) assay

The *V. cholerae* isolates were treated with the heavy metals as described in the 2D-GE Analysis section. Total RNA was extracted, qRT-PCR was performed, the relative expression of representative genes were calculated according to the method described in our recent reports (Zhu et al., 2020; Shan et al., 2022; Yan et al., 2022). Primers were synthesized by Biotech Bioengineering (Shanghai, China). The untreated bacterial culture was used as a negative control.

### Data analysis

In this study, all tests were performed in triplicate. The data were analyzed using the SPSS software version 17.0 (SPSS Inc., Armonk, NY, USA). The one-way analysis of variance (ANOVA) followed by appropriate post-hoc test (Tukey) was used to determine significant difference (*p* < 0.05).

## Results

### MICs of the heavy metals against the *Vibrio cholerae* isolates

The *V. cholerae* J9-62, Q6-10, and N9-4 isolates of aquatic animal origins showed different heavy metal tolerant profiles (Chen et al., 2021). The tolerance of *V. cholerae* J9-62 to Pb^2+^, *V. cholerae* Q6-10 to Cd^2+^ and Zn^2+^; and *V. cholerae* N9-4 to Ni^2+^ was chosen for the further analyses in this study (Supplementary Table S1).

The MICs of the heavy metals were determined, and the results showed that when compared to the quality control strain *E. coli* K-12, *V. cholerae* J9-62 was tolerant to Pb^2+^ with MIC value of 3,200 μg/mL; *V. cholerae* Q6-10 to Cd^2+^, and Zn^2+^ with MIC values of 400 μg/mL, and 800 μg/mL, respectively; and *V. cholerae* N9-4 to Ni^2+^ with MIC value of 400 μg/mL (Supplementary Table S2). These results underlined the high heavy metal tolerance of the *V. cholerae* isolates of the aquatic animal origins.

### Growth of the *Vibrio cholerae* isolates under different concentrations of the heavy metals

**Figure 1:**
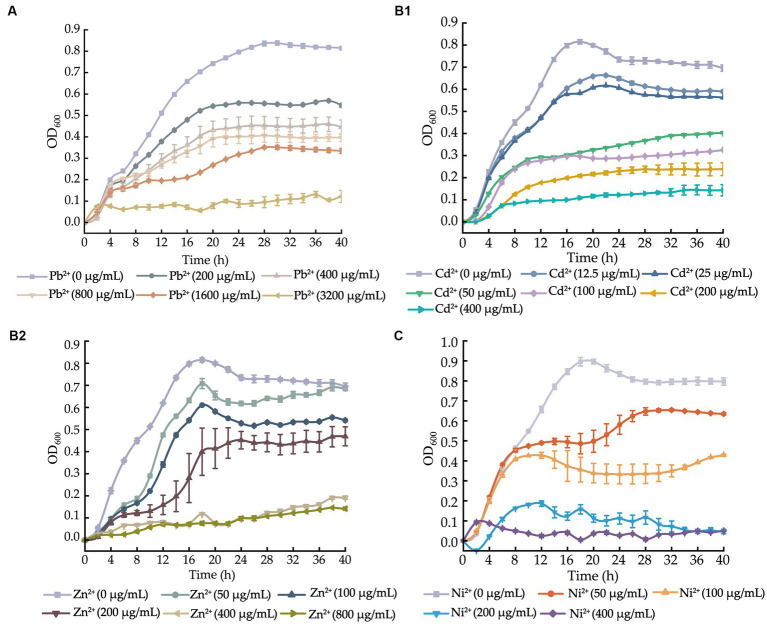
Growth curves of the *V. cholerae* isolates incubated in the TSB medium supplemented with different concentrations of heavy metals at 37°C. **(A–C)** *V. cholerae* J9–62 **(A)**, Q6–10 **(B1,B2)**, and N9–4 **(C)** isolates, respectively.

Based on the above results, we determined growth curves of *V. cholerae* J9-62, Q6-10, and N9-4 isolates in the TSB medium supplemented with different concentrations of the heavy metals at 37°C, and the results are shown in Figure 1.

After supplemented with the Pb^2+^ (3200–200 μg/mL), we observed that the growth of *V. cholerae* J9-62 was greatly inhibited at 3200 μg/mL of Pb^2+^. Upon the decreased Pb^2+^ concentrations (1600–400 μg/mL), the increased growth of *V. cholerae* J9-62 was observed. The maximum biomass was detected at 200 μg/mL of Pb^2+^ with the OD_600_ value of 0.544, as compared to the control group (Figure 1A).

Similarly, growth curves of *V. cholerae* Q6-10 were determined under the Cd^2+^ (400–12.5 μg/mL) or Zn^2+^ (800–50 μg/mL) conditions. As shown in Figure 1B1, *V. cholerae* Q6-10 was highly inhibited at 400 and 200 μg/mL of Cd^2+^, while little growth was observed at 100 and 50 μg/mL of Cd^2+^, respectively. *V. cholerae* Q6-10 was slightly inhibited at 25 and 12.5 μg/mL of Cd^2+^ with the maximum OD_600_ values of 0.578 and 0.636, respectively. In addition, under the Zn^2+^ (800–50 μg/mL) treatment conditions, the growth of *V. cholerae* Q6-10 was fully inhibited at 800 and 400 μg/mL of Zn^2+^, respectively. The isolate still grew poorly at 200 μg/mL of Zn^2+^, whereas slight inhibition was observed at 50 μg/mL of Zn^2+^ with the maximum OD_600_ values of 0.625, as compared to the control group (Figure 1B2).

Growth curves of *V. cholerae* N9-4 were determined under the Ni^2+^ (400–50 μg/mL) conditions. As shown in Figure 1C, the growth of *V. cholerae* N9-4 was highly inhibited at 400 μg/mL and 200 μg/mL of Ni^2+^, respectively. The isolate still grew poorly at 100 μg/mL of Ni^2+^, whereas a slight decrease in growth was observed at 50 μg/mL of Ni^2+^, with the maximum OD_600_ value of 0.624, as compared to the control group (Figure 1C).

Taken together, based on these results, the 200 μg/mL of Pb^2+^ for *V. cholerae* J9-62; the 12.5 μg/mL of Cd^2+^ or 50 μg/mL of Zn^2+^ for *V. cholerae* Q6-10; and the 50 μg/mL of Ni^2+^ for *V. cholerae* N9-4 were chosen as the treatment conditions in the further analyses, respectively.

### Changes in cell morphological structure of the *Vibrio cholerae* isolates under the heavy metal stresses

**Figure 2:**
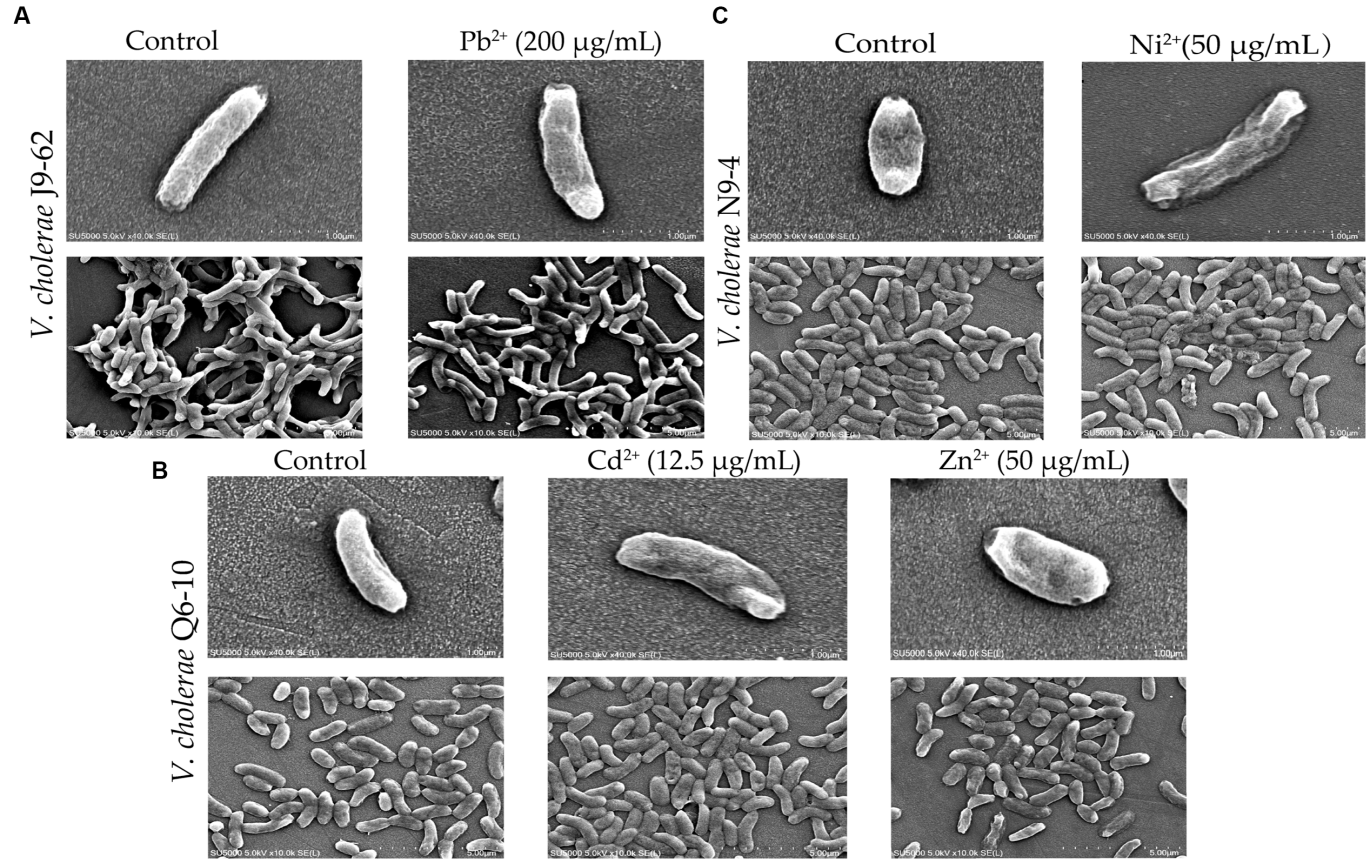
The SEM assay of cell surface structure of the *V. cholerae* isolates under the heavy metal stresses. *V. cholerae* J9–62, Q6–10, and N9–4 isolates were treated with 200 μg/mL of Pb^2+^ **(A)** 12.5 μg/mL of Cd^2+^ **(B)** or 50 μg/mL of Zn^2+^ **(B)** and 50 μg/mL of Ni^2+^ **(C)** for 2 h, respectively. The untreated bacterial cells were used as controls.

As shown in Figure 2, bacterial cells in the control groups were flat, intact and rod-shaped (Figures 2A1,B1,C1), however, after being treated with the Pb^2+^ (200 μg/mL) for 2 h, the cell surface of *V. cholerae* J9-62 was slightly deformed, with obvious depressions and wrinkles (Figure 2A2). Similarly, after treated with the Cd^2+^ (12.5 μg/mL), the cell surface of *V. cholerae* Q6-10 slightly folded (Figure 2B2); and the same case was observed under the Zn^2+^ (50 μg/mL) treatment (Figure 2B3). For *V. cholerae* N9-4, the treatment with the Ni^2+^ (50 μg/mL) also led to the bacterial cell surface slightly shrank (Figure 2C2). Additionally, we observed that the extended treatment time (≥4 h) resulted in the bacterial cell broke (Figures not shown).

### Survival of the *Vibrio cholerae* isolates under the heavy metal stresses

Based on the above results, we also examined fatality rates of the *V. cholerae* isolates under the heavy metal stresses, and the results are shown in Supplementary Table S2. Approximately 42.75% of *V. cholerae* J9-62 cells could not survive after being treated with the Pb^2+^ (200 μg/mL) for 2 h. Similarly, the fatality rates of *V. cholerae* Q6-10 under the Cd^2+^ (12.5 μg/mL), or Zn^2+^ (50 μg/mL) stresses were 27.98%, or 29.70%, respectively, while that of *V. cholerae* N9-4 under the Ni^2+^(50 μg/mL) stress was 34.42%. These results highlighted that the *V. cholerae* isolates were capable of surviving under the heavy metal stresses, with the fatality rates ranging from 27.98 to 42.75%.

### The effects of the heavy metal stresses on cell membrane permeability and fluidity, and cell surface hydrophobicity of the *Vibrio cholerae* isolates

**Figure 3:**
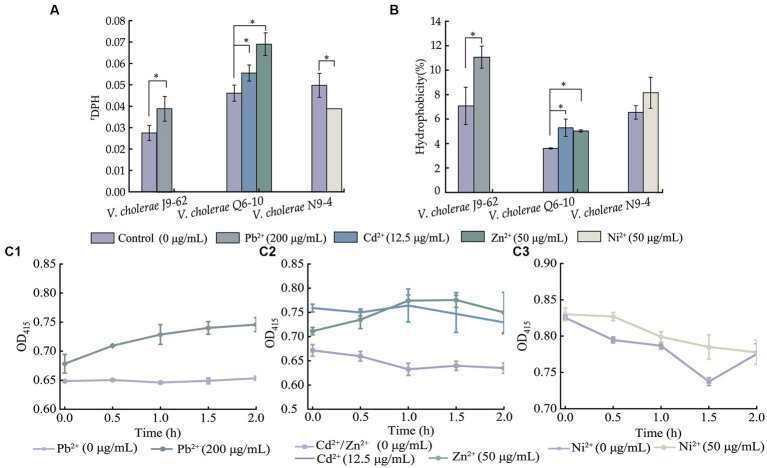
The cell membrane fluidity **(A)**, surface hydrophobicity **(B)**, and inner cell membrane permeability **(C)** of the *V. cholerae* isolates under the heavy metal stresses. *V. cholerae* J9–62, Q6–10, and N9–4 isolates **(C1–C3)** grown in the TSB medium were treated with the heavy metals at 37°C for 2 h, respectively. *p < 0.05.

Bacterial cell membrane fluidity and permeability, and cell surface hydrophobicity are key parameters of cell membrane that undergoes quick adaptation to environmental changes (Rogers et al., 2021). Therefore, based on the above results, we further investigated the effects of the heavy metal stresses on the *V. cholerae* cell membrane integrity (Figure 3).

As shown in Figure 3A, when compared to the control group, the cell membrane fluidity of *V. cholerae* J9-62 was significantly reduced by 1.422–fold after being treated with the Pb^2+^ (200 μg/mL) for 2 h (*p <* 0.05). Similarly, the cell membrane fluidity of *V. cholerae* Q6-10 was also reduced by 1.204–fold and 1.495–fold under the treatment with Cd^2+^ (12.5 μg/mL) and Zn^2+^ (50 μg/mL) stresses, respectively (*p <* 0.05). Conversely, the treatment with the Ni^2+^ (50 μg/mL) increased the cell membrane fluidity of *V. cholerae* N9-4 by 1.285–fold (*p <* 0.05).

As shown in Figure 3B, as compared with the control group, cell surface hydrophobicity of *V. cholerae* J9-62 was significantly enhanced by 1.563–fold after treated with the Pb^2+^ for 2 h (*p <* 0.05). Similarly, an increase by 1.468–fold, and 1.397–fold was observed in cell surface hydrophobicity of *V. cholerae* Q6-10 after treated with the Cd^2+^, and Zn^2+^, respectively (*p <* 0.05). In addition, there was no significant change in cell surface hydrophobicity of *V. cholerae* N9-4 after treated with the Ni^2+^ (50 μg/mL) (*p* > 0.05).

As shown in Figure 3C, as compared to the control group, after being treated with the Pb^2+^, the inner cell membrane permeability of *V. cholerae* J9-62 was significantly increased (*p <* 0.05) (Figure 3A). The similar cases were observed after *V. cholerae* Q6-10 being treated with the Cd^2+^ or Zn^2+^, respectively (Figure 3B). Additionally, there was no significant change in the inner membrane permeability of *V. cholerae* N9-4 under the treatment with Ni^2+^ (Figure 3C).

Taken together, the results demonstrated that the Pb^2+^ (200 μg/mL), Cd^2+^ (12.5 μg/mL), and Zn^2+^ (50 μg/mL) stresses significantly decreased cell membrane fluidity of *V. cholerae* J9-62, and Q6-10 isolates (*p <* 0.05), but increased the bacterial cell surface hydrophobicity, and inner membrane permeability, respectively (*p <* 0.05). Exceptionally, the Ni^2+^ (50 μg/mL) stress only significantly increased the cell membrane fluidity of *V. cholerae* N9-4 (*p <* 0.05).

### Distinct secretomes of the *Vibrio cholerae* isolates under the heavy metal stresses

**Figure 4:**
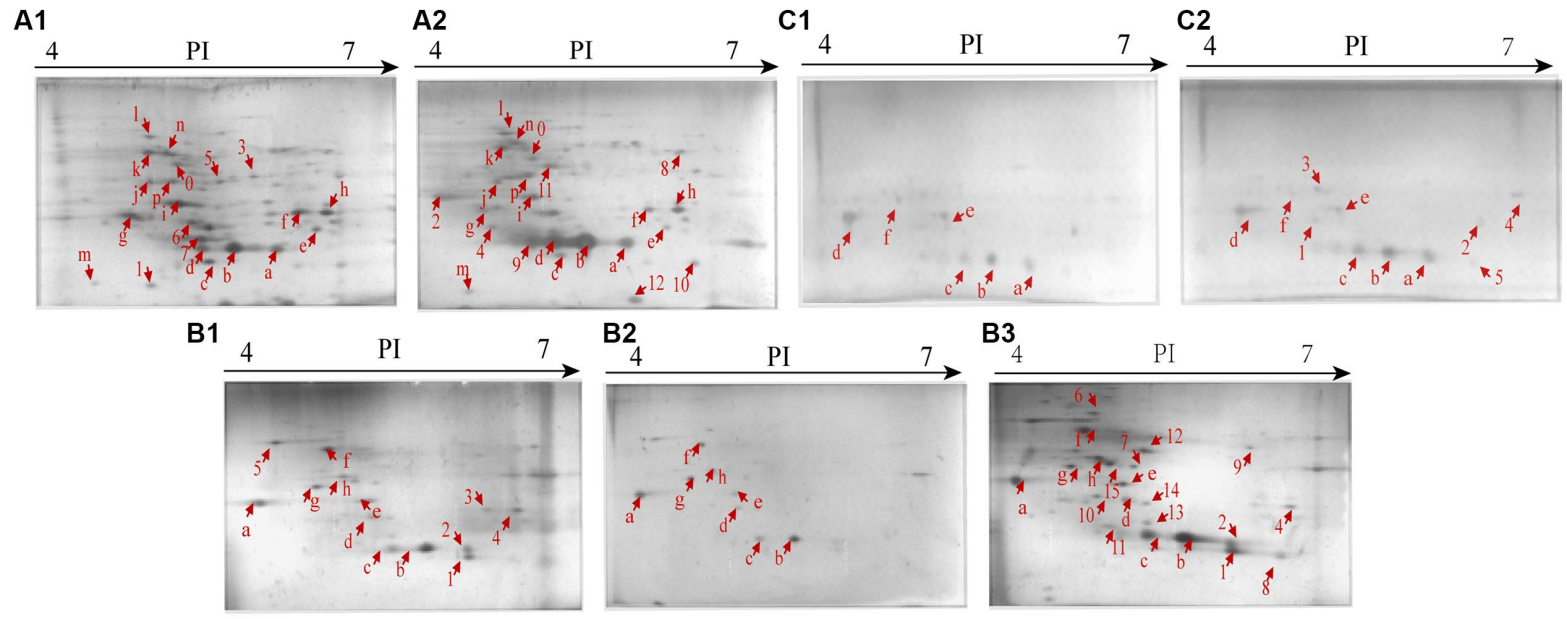
Secretomic profiles of the *V. cholerae* isolates under the heavy metal stresses by the 2D-GE analysis. **(A1,A2)** *V. cholerae* J9–62 incubated in the TSB medium without and with the Pb^2+^(200 μg/mL) treatment, respectively. **(B1–B3)** *V. cholerae* Q6–10 without and with the Cd^2+^(12.5 μg/mL) or Zn^2+^ (50 μg/mL) treatment, respectively. **(C1,C2)** *V. cholerae* N9–4 without and with the Ni^2+^ (50 μg/mL) treatment, respectively.

Secretomes of the *V. cholerae* isolates under the heavy metal stresses were obtained by the 2D-GE analysis (Figures 4A–C). Secretome patterns produced by three independent 2D-GE experiments of each isolate were consistent (Figures not shown). Comparative secretomic analysis revealed that *V. cholerae* J9-62, Q6-10, and N9-4 isolates secreted 30 common proteins (marked with different red letters, Figure 4 and Supplementary Table S3), and 32 differential proteins (marked with different red numbers, Figure 4 and Supplementary Table S4) under the Pb^2+^ (200 μg/mL), Cd^2+^ (12.5 μg/mL) and Zn^2+^ (50 μg/mL), and Ni^2+^ (50 μg/mL) stresses, respectively, as compared to the control groups. Amino acid sequences of each of these extracellular proteins were further determined by the LC–MS/MS analysis.

Remarkably, some differential extracellular proteins of *V. cholerae* J9-62, N9-4, and Q6-10 isolates were identified (*n* = 5 to 10) under the heavy metal stresses. For example, after treated with the Pb^2+^ (200 μg/mL) for 2 h, *V. cholerae* J9-62 secreted seven more extracellular proteins than the control group; *V. cholerae* Q6-10 secreted ten more, but five less extracellular proteins under the Zn^2+^ (50 μg/mL), and Cd^2+^(12.5 μg/mL) stresses, respectively; and *V. cholerae* N9-4 secreted five more extracellular proteins under the Ni^2+^ (50 μg/mL) stress.

### Identification of differential extracellular proteins of the *Vibrio cholerae* isolates under the heavy metal stresses

A total of 32 differential extracellular proteins were identified using the LC–MS/MS analysis (Supplementary Table S4). Of these, 20 extracellular proteins were grouped into three main Gene Onotology (GO) categories, whereas 12 had unknown function (Figure not shown).

The Pb^2+^ (200 μg/mL), Zn^2+^ (50 μg/mL), and Ni^2+^ (50 μg/mL) stresses increased extracellular protein secretion of *V. cholerae* J9-62, Q6-10, and N9-4 isolates, respectively (Figure 4). For example, approximately seven extracellular proteins were secreted by *V. cholerae* J9-62 under the Pb^2+^ (200 μg/mL) stress, including a gntP family permease (Spot A-8), a hydroxyacylglutathione hydrolase (Spot A-9), a Thiol: disulfide interchange protein (Spot A-10), an enolase (Spot A-11), a leucine aminopeptidase (Spot A-12), a porin_4 domain-containing protein (Spot A-2), and a DUF91 domain-containing protein (Spot A-4) (Figure 4A2).

Approximately ten extracellular proteins were secreted by *V. cholerae* Q6-10 under the Zn^2+^ (50 μg/mL) stress, e.g., a putrescine-binding periplasmic protein (Spot B-7), a S8 family peptidase (Spot B-9), and an outer membrane protein A (OmpA) (Spot B-10) (Figure 4B3), while approximately five differential extracellular proteins were identified from secretomic profiles of *V. cholerae* N9-4 under the Ni^2+^(50 μg/mL) stress, e.g., a maltodextrin-binding protein (Spot C-1), a flagellin (Spot C-3), and a periplasmic thiosulfate-binding protein (Spot C-4) (Figure 4C2).

Conversely, under the Cd^2+^(12.5 μg/mL) stress, *V. cholerae* Q6-10 secreted less extracellular proteins (*n* = 5) than those in the control group (Figure 4B2).

### Identification of differential intracellular proteins (DIPs) in the *Vibrio cholerae* isolates under the heavy metal stresses

#### Identification of DIPs in *Vibrio cholerae* J9-62 under the Pb^2+^ stress

**Figure 5:**
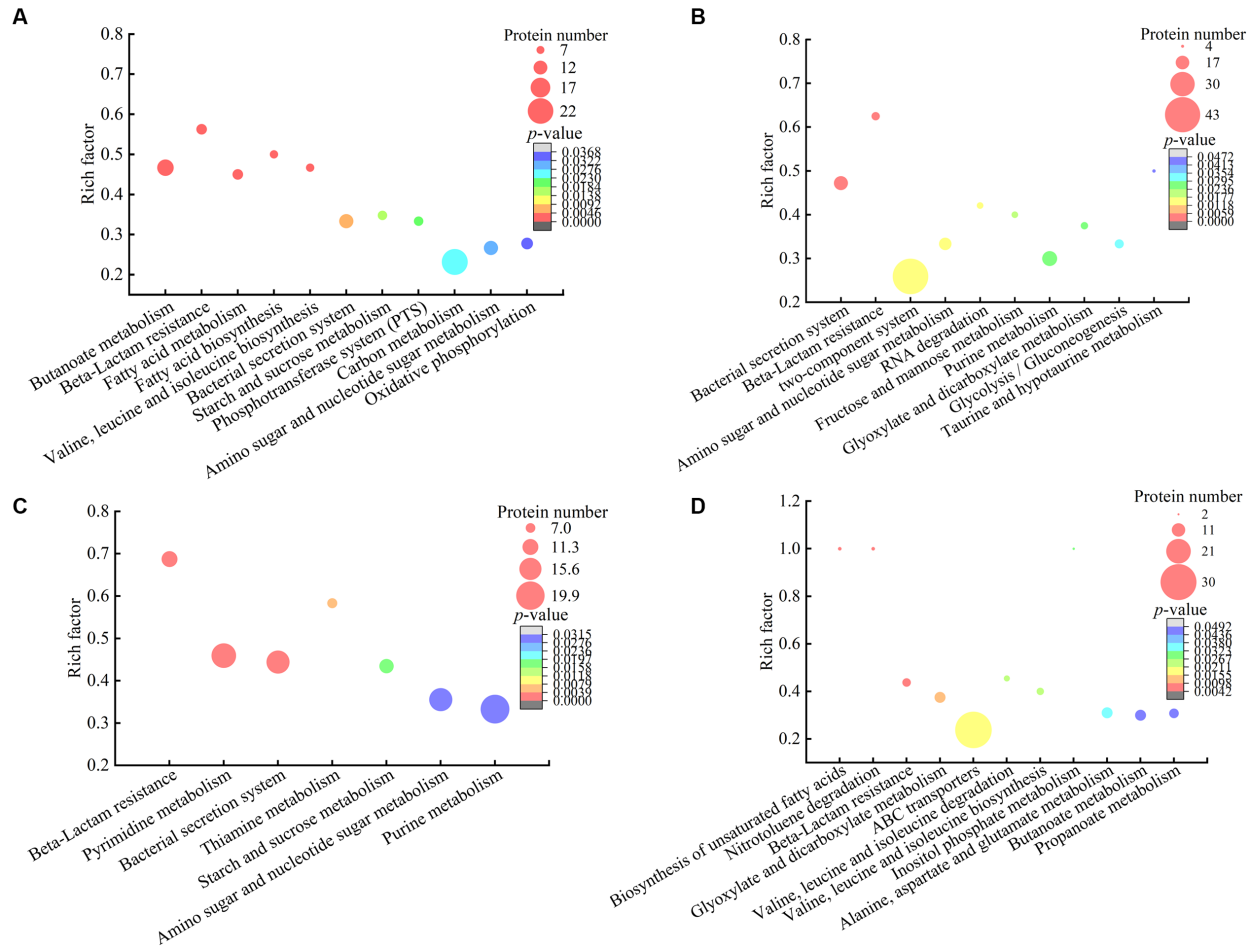
The metabolic pathways with significant enrichment of DIP produced by the *V. cholerae* isolates. **(A–D)** *V. cholerae* J9–62, Q6–10, and N9–4 under the Pb^2+^; Cd^2+^or Zn^2+^; and Ni^2+^ stresses, respectively.

A total of 417 DIPs in *V. cholerae* J9-62 under the Pb^2+^ (200 μg/mL) stress for 2 h were identified by the LC–MS/MS analysis, as compared to the control group. Of these, 316 DIPs were grouped into GO categories, whereas 101 DIPs had unknown function. The most abundant GO term of the DIPs was the cellular process (79.75%, 252/316), followed by metabolic processes (75.95%, 240/316), and single-organism process (73.10%, 231/316) (Figure 5A).

The DIPs were significantly enriched in eleven metabolic pathways, including the butanoate metabolism, beta-lactam resistance, fatty acid metabolism, fatty acid biosynthesis, valine, leucine and isoleucine biosynthesis, bacterial secretion system, starch and sucrose metabolism, phosphotransferase system (PTS), carbon metabolism, amino sugar and nucleotide sugar metabolism, and oxidative phosphorylation (*p <* 0.05).

For example, in the beta-lactam resistance, the DIPs involved in heavy metal tolerance were found in *V. cholerae* J9-62 under the Pb^2+^ stress, e.g., an efflux transporter outer membrane subunit, a putative multidrug resistance protein, an ATP-binding protein, and a Co/Zn/Cd efflux system membrane fusion protein. Metal ion transportation occurs in active mode by ATP-binding cassette (ABC) transporters in bacterial cells (Mitra et al., 2021). It has been reported that metal exclusion occurred *via* resistance-nodulation cell division proteins across the proton gradient in *E. coli*, *Candida albicans*, and *Pseudomonas putida* to reduce cytotoxicity of heavy metals (Mitra et al., 2021).

In the butanoate metabolism, the expression of a glutathione peroxidase and a succinate-semialdehyde dehydrogenase was also induced in *V. cholerae* J9-62 under the Pb^2+^ stress. The glutathione peroxidase is an important antioxidant enzyme. The elevation of glutathione after the Pb treatment of *Rhodotorula mucilaginosa* contributed to the enhanced detoxification of Pb (Chen et al., 2022). The succinate semialdehyde dehydrogenase directly participates in the formation of α-ketoglutarate that is an essential metabolite involved in anti-oxidative defense, energy production, signaling modules, and genetic modification in cells (Legendre et al., 2020).

In energy metabolic pathways such as the carbon metabolism, starch and sucrose metabolism, oxidative phosphorylation, as well as amino sugar and nucleotide sugar metabolism, the expression of some DIPs was enriched under the Pb^2+^ stress, e.g., a glyceraldehyde 3-phosphate dehydrogenase (GAPDH), a glucose-6-phosphate isomerase (GPI), an acetyl-CoA acetyltransferase, and a putative NADH oxidase. The coordination of these pathways may have provided critical material flux and energy for cell processes toward the Pb stress in *V. cholerae* J9-62.

Taken together, under the Pb^2+^ stress, *V. cholerae* J9-62 developed multiple defensive strategies for reducing cytotoxicity of the Pb: (1) induced the expression of transportation, efflux, and secretion systems-related proteins; (2) triggered the expression of antioxidative defense enzymes; (3) elicited the biosynthesis of hydrophobic amino acids; and (4) enriched the energy metabolism-related proteins.

#### Identification of DIPs in *Vibrio cholerae* Q6-10 under the Cd^2+^ stress

A total of 521 DIPs were identified in *V. cholerae* Q6-10 under the Cd^2+^ (12.5 μg/mL) stress for 2 h by the LC–MS/MS analysis, as compared to the control group. Of these, 427 DIPs were grouped into GO categories, whereas 94 DIPs had unknown function. The most abundant GO term of the DIPs was the cellular process (77.98%, 333/427), followed by metabolic processes (72.83%, 311/427), and single-organism process (64.87%, 277/427) (Figure 5B).

The DIPs were significantly enriched in bacterial secretion system, beta-lactam resistance, two-component system, amino sugar and nucleotide sugar metabolism, RNA degradation, fructose and mannose metabolism, purine metabolism, glyoxylate and dicarboxylate metabolism, glycolysis/gluconeogenesis, and taurine and hypotaurine metabolism (*p <* 0.05). Three of these metabolic pathways (bacterial secretion system, beta-lactam resistance, as well as amino sugar and nucleotide sugar metabolism) were also altered in the Pb^2+^-induced *V. cholerae* J9-62.

For example, in the bacterial secretion system, the DIPs related to heavy metal Cd^2+^ resistance were identified, including type VI secretion system (T6SS) ATPase TssH, and IcmF. T6SS can secret various metal-binding proteins to promote bacterial survival in harmful environments through metal ion acquisition (Wang et al., 2021; Dang et al., 2022). For instance, Hu et al. reported that T6SS contributed to zinc stress resistance in a BaeSR system-dependent manner (Hu et al., 2020).

In the beta-lactam resistance, the expression of a multidrug efflux resistance-nodulation-cell division (RND) transporter permease subunit VexB, an oligopeptide ABC transporter, an ATP-binding protein, and a vibriobactin export RND transporter periplasmic adaptor subunit VexG was induced under the Cd^2+^ stress in *V. cholerae* Q6-10. The RND family protein was able to efflux Cd from the cytoplasm to the periplasm in *Bacillus vietamensis* thereby alleviating its toxicity (Yu et al., 2020).

In the fructose and mannose metabolism, the expression of an exopolysaccharide (EPS) biosynthesis protein was identified. Fang et al. (2022) reported that *Cupriavidus nantongensis* X1^T^ strain produced EPS under the stress of Cd^2+^, which immobilized Cd^2+^ to protect the cells against the Cd^2+^ toxicity (Fang et al., 2022). Extracellular adsorption was the main pathway for microorganisms to remove Cd^2+^ from media to reduce its cytotoxicity (Heidari and Panico, 2020).

Taken together, under the Cd^2+^ (12.5 μg/mL) stress, *V. cholerae* Q6-10 developed multiple strategies to efficiently alleviate the Cd cytotoxicity: (1) induced the expression of transportation and efflux of multidrug efflux RND transporters; (2) elicited the expression of proteins related to the regulation of glutathione metabolism; (3) triggered the accumulation of taurine; (4) induced the expression of the EPS biosynthesis proteins; and (5) enriched the energy metabolism-related proteins.

#### Identification of DIPs in *Vibrio cholerae* Q6-10 under the Zn^2+^ stress

A total of 655 DIPs were identified in *V. cholerae* Q6-10 under the Zn^2+^ (50 μg/mL) stress for 2 h by the LC–MS/MS analysis, as compared to the control group. Of these, 498 DIPs were grouped into GO categories, whereas 157 DIPs had unknown function. The most abundant GO term of the DIPs was the cellular process (79.92%, 398/498), followed by metabolic processes (79.52%, 396/498), and catalytic activity (68.88%, 343/498) (Figure 5C).

The DIPs were significantly enriched in beta-lactam resistance, pyrimidine metabolism, bacterial secretion system, thiamine metabolism, starch and sucrose metabolism, amino sugar and nucleotide sugar metabolism, and purine metabolism (*p <* 0.05). Three of these metabolic pathways (bacterial secretion system, beta-lactam resistance, as well as amino sugar and nucleotide sugar metabolism) were also altered in the Pb^2+^-induced *V. cholerae* J9-62, and the Cd^2+^-induced *V. cholerae* Q6-10. Moreover, the starch and sucrose metabolism was also altered in the Pb^2+^-induced *V. cholerae* J9-62.

For example, in the beta-lactam resistance, the expression of the DIPs involved in the Zn^2+^ resistance was identified in *V. cholerae* Q6-10, including a multidrug efflux RND transporter permease subunit VexB, a multidrug efflux RND transporter periplasmic adaptor subunit VexA, and a peptide ABC transporter. Bacterial drug-efflux transporters acted synergistically as diffusion barriers of cellular membranes to protect cells from heavy metals and toxic metabolites (Zgurskaya et al., 2022).

In the amino sugar and nucleotide sugar metabolism, the expression of an iron–sulfur cluster assembly protein CyaY, and an iron–sulfur cluster carrier protein was induced in *V. cholerae* Q6-10 under the Zn^2+^ stress. The metal-binding domain of iron–sulfur proteins functioned to entrap metallic elements inside the cells (Huang et al., 2019). Extracellular adsorption and intracellular accumulation were found to be the main pathways for microorganisms to remove Zn^2+^ from media to get rid of the Zn^2+^ toxicity (Vargas-García Mdel et al., 2012). In addition, amino sugar and nucleotide sugar metabolism, and starch and sucrose metabolism likely provided energy for cellular activities and maintained stability of the bacterial cell under the Zn^2+^ stress (Lee et al., 2016).

Taken together, under the Zn^2+^ (50 μg/mL) stress, *V. cholerae* Q6-10 employed multiple strategies to efficiently alleviate its cytotoxicity: (1) induced the expression of multidrug efflux RND transporters, and ABC transporters; (2) triggered the expression of extracellular adsorption and intracellular accumulation-related proteins; (3) elicited the expression of stress-related proteins; and (4) enriched the energy metabolism-related proteins.

#### Identification of DIPs in *Vibrio cholerae* N9-4 under the Ni^2+^ stress

A total of 441 DIPs were identified in *V. cholerae* N9-4 under the Ni^2+^ (50 μg/mL) stress for 2 h by the LC–MS/MS analysis, as compared to the control group. Of these, 378 DIPs were grouped into GO categories, whereas 63 DIPs had unknown function. The most abundant GO term of the DIPs was metabolic process (78.31%, 296/378), followed by catalytic activity (70.37%, 266/378), and single-organism process (65.87%, 249/378) (Figure 5D).

The DIPs were significantly enriched in the biosynthesis of unsaturated fatty acids, nitrotoluene degradation, beta-lactam resistance, glyoxylate and dicarboxylate metabolism, ABC transporters, valine, leucine and isoleucine degradation, valine, leucine and isoleucine biosynthesis, inositol phosphate metabolism, alanine, aspartate and glutamate metabolism, butanoate metabolism, and propanoate metabolism (*p <* 0.05). Of these, the bacterial secretion system was also altered in the Pb^2+^-induced *V. cholerae* J9-62, Cd^2+^-induced *V. cholerae* Q6-10, and Zn^2+^-induced *V. cholerae* Q6-10; the butanoate metabolism, and valine, leucine and isoleucine biosynthesis were changed in the Pb^2+^-induced *V. cholerae* J9-62 as well; the glyoxylate and dicarboxylate metabolism was also altered in the Cd^2+^-induced *V. cholerae* Q6-10.

For instance, in the ABC transporters, the expression of an ABC transporter substrate-binding protein, an arginine ABC transporter, a sulfate ABC transporter substrate-binding protein, a peptide/nickel transport system substrate-binding protein, and a cysteine/glutathione ABC transporter permease was induced in *V. cholerae* N9-4 under the Ni^2+^ stress. ABC transporters can secret and excret foreign substances across cell membrane to maintain cellular homeostasis (Fan et al., 2023). They can export the cations as a metal-glutathione complex to reduce cytotoxicity of certain metals (Pearson and Cowan, 2021).

In the glyoxylate and dicarboxylate metabolism, the expression of a short-chain dehydrogenase/reductase (SDR) family oxidoreductase, a dihydrolipoamide dehydrogenase (DLD), and a bifunctional glutamine synthetase adenylyltransferase/adenylyl-removing enzyme was induced in *V. cholerae* N9-4 under the Ni^2+^ stress. For example, aldehyde reductases were identified as critical enzymes for catalyzing the detoxification reactions of aldehydes in *Saccharomyces cerevisiae* (Wang et al., 2019).

Taken together, under the Ni^2+^ (50 μg/mL) stress, *V. cholerae* N9-4 developed multiple strategies to efficiently reduce its cytotoxicity: (1) induced the expression of efflux pump RND transporters, ABC transporters, and T6SS-associated proteins; (2) triggered the expression of extracellular adsorption-associated proteins; (3) elicited the expression of stress-related proteins; and (4) triggered the glyoxylate and dicarboxylate metabolism pathways.

### The main defensive strategies of the *Vibrio cholerae* isolates under the Pb^2+^, Cd^2+^, Zn^2+^, and Ni^2+^ stresses

The comparative secretomic and proteomic analyses revealed common strategies developed by the *V. cholerae i*solates under different heavy metal (Pb^2+^, Cd^2+^, Zn^2+^, and Ni^2+^) stresses, such as the activation of efflux pump RND transporters, ABC transporters and metal chelators for effluxing; the expression of glutathione peroxidase for reducing oxidative stress damage; the biosynthesis of EPS for extracellular biosorption and sequestration; and the activation of energy metabolism-related pathways.

**Figure 6:**
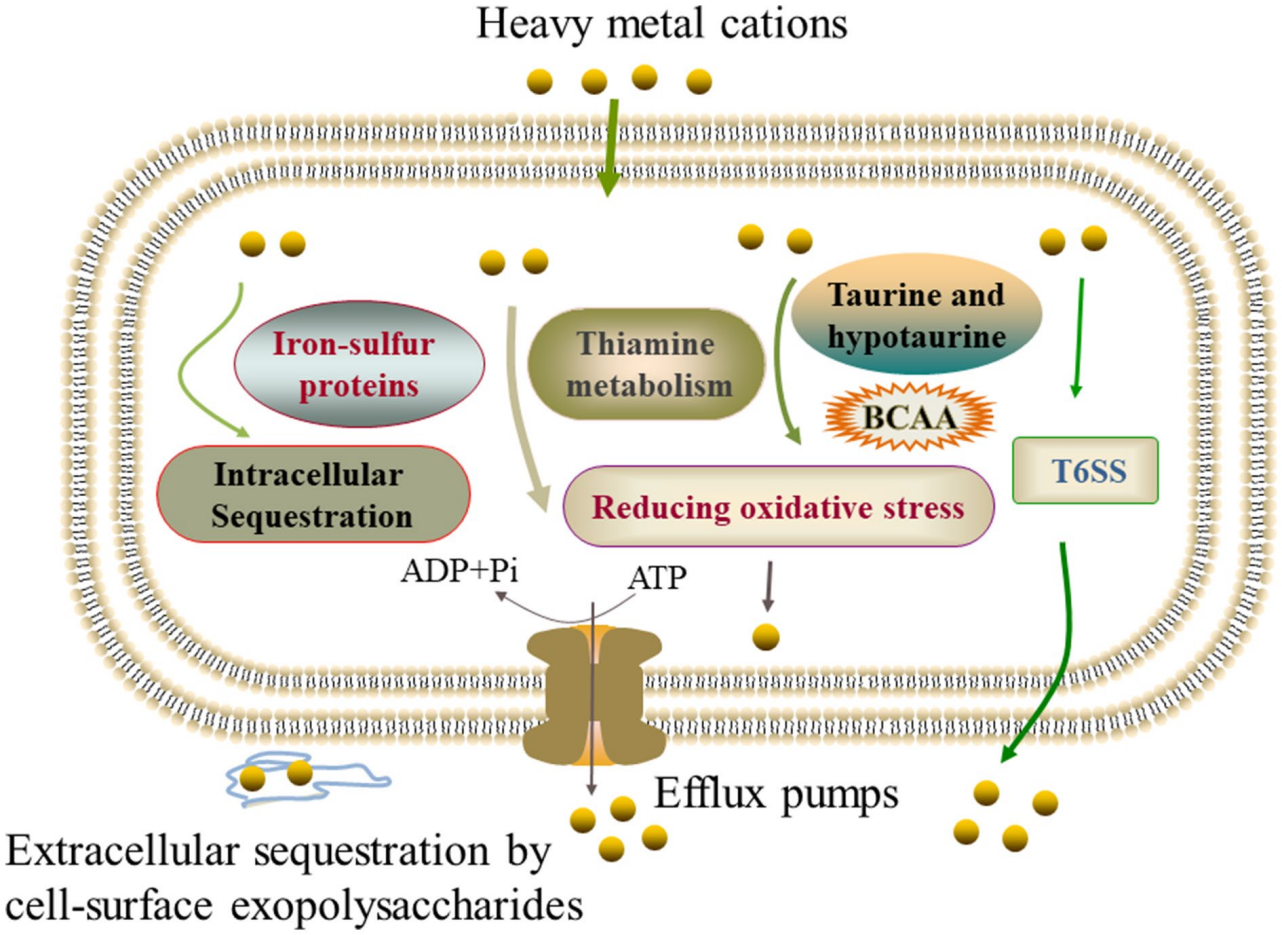
The mechanistic diagram of the *V. cholerae* tolerance toward the heavy metals Pb^2+^, Cd^2+^, Zn^2+^, and Ni^2+^ stresses. BCAA, branched-chain amino acid, e.g., isoleucine, valine, and leucine.

Notably, different strategies were also found in the *V. cholerae* isolates to cope with different heavy metal stresses. For example, *V. cholerae* J9-62 reduced the Pb^2+^ cytotoxicity by inducing the biosynthesis of hydrophobic amino acids; *V. cholerae* Q6-10 mitigated the Cd^2+^ damage by inducing taurine accumulation; *V. cholerae* Q6-10 reduced the Zn^2+^ hazard by elicting the iron–sulfur protein expression and thiamin biosynthesis; and *V. cholerae* N9-4 reduced the Ni^2+^ cytotoxicity by triggering the expression of T6SS-associated proteins (Figure 6).

### The effects of the heavy metal stresses on putative virulence-associated proteins in the *Vibrio cholerae* isolates

In this study, among the 32 identified differential extracellular proteins, four virulence-associated proteins were secreted by the *V. cholerae* J9-62, N9-4, and Q6-10 isolates. Among the 2,034 identified DIPs produced by the *V. cholerae* isolates, approximately 108 were directly or indirectly involved in the virulence of pathogenic bacteria, such as adhesion, invasion, damage of host cells, and regulation of virulence (Supplementary Table S5).

For example, under the Zn^2+^ (50 μg/mL) stress, *V. cholerae* Q6-10 secreted more extracellular virulence-associated proteins (*n* = 3) than the control group, including an OmpA (Spot B-10), a high-affinity zinc uptake system protein ZnuA (Spot B-13), and a PrkA serine protein kinase (Spot B-14). It has been reported that OmpA was a highly multifunctional protein required for bacterial virulence in *Pseudomonas aeruginosa* (Paulsson et al., 2021). PrkA is required for cell wall stress responses, and virulence in *Listeria monocytogenes* (Kelliher et al., 2021).

Approximately 89 intracellular virulence-associated proteins were produced by *V. cholerae* J9-62, and Q6-10 isolates under the Pb^2+^ (200 μg/mL) (*n* = 25), Cd^2+^ (12.5 μg/mL) (*n* = 26), and Zn^2+^ (50 μg/mL) (*n* = 38) stresses (Supplementary Table S5), e.g., a T6SS-associated forkhead-associated (FHA) domain protein TagH (Spot 558), a transmembrane regulator ToxS (Spot 980), and a cholix toxin (Spot 927). For example, it has been reported that the TagH regulated the hemolytic activity and virulence of *V. cholerae* (Wang G. et al., 2022).

Under the Ni^2+^ (50 μg/mL) stress, *V. cholerae* N9-4 expressed more intracellular virulence-associated proteins (*n* = 20) than the control group, e.g., a NADH-dependent flavin oxidoreductase (NFOR) (Spot 729), a T6SS protein VasJ (Spot 1147), a transcriptional activator HlyU (Spot 1046), and an OmpV (Spot 812). For instance, Xie et al. (2021) reported that NFOR was involved in the pathogenicity of *Mycoplasma hyopneumoniae*. HlyU is a transcriptional regulator essential for the virulence of *Vibrio vulnificus* (Lee et al., 2019). OmpV played a vital role in the pathogenesis of *Salmonella typhimurium*, including the adhesion and invasion to intestinal epithelial cells (Kaur et al., 2021).

These results highlighted that a number of putative virulence-associated proteins (*n* = 112) were differently produced and or secreted by the *V. cholerae* isolates under the heavy metal stresses, implying an increased health risk of the *V. cholerae* isolates in aquatic products triggered by the heavy metal stresses.

### The effects of the heavy metal stresses on putative resistance-associated proteins in the *Vibrio cholerae* isolates

In this study, one differential extracellular protein, and approximately 55 DIPs involved in bacterial resistance were identified in the *V. cholerae* isolates under the heavy metal stresses (Supplementary Table S6).

For instance, there were 35 intracellular resistance-associated proteins produced by *V. cholerae* J9-62, and Q6-10 isolates under the Pb^2+^ (200 μg/mL) (*n* = 10), Cd^2+^ (12.5 μg/mL) (*n* = 6), and Zn^2+^ (50 μg/mL) (*n* = 19) stresses, e.g., a multidrug transporter AcrB (Spot 563), and a tetR family transcriptional regulator (Spot 1133). Under the Ni^2+^ (50 μg/mL) stress, *V. cholerae* N9-4 produced more intracellular resistance-associated proteins (*n* = 23) than the control group, e.g., a lytic transglycosylases (Spot 1115), and a putative glutathione S-transferase (Spot 1058).

In addition, to confirm the identified proteins by the 2D-GE and LC–MS/MS methods, the qRT-PCR was carried out to examine the expression of randomly chosen differential proteins. The obtained results were in agreement with those by the secretomic and proteomic analyses in this study (Supplementary Table S7 and Supplementary Figure S1).

## Discussion

*V. cholerae* is frequently isolated from aquatic products (Xu et al., 2019; Fu et al., 2020; Chen et al., 2021). China is the largest producer, exporter, and consumer of aquatic products worldwide. Thus, it is of great significance to investigate molecular mechanisms of *V. cholerae* of aquatic animal origins toward the heavy metal stresses in order to effectively control the pathogen in aquatic products.

The MIC is currently the best available parameter to reflect the effectiveness of an antibiotic or heavy metal against bacterial strains (Kowalska-Krochmal and Dudek-Wicher, 2021). In this study, the MIC values of Cd^2+^, and Zn^2+^ was 400 μg/mL, and 800 μg/mL against *V. cholerae* Q6-10, respectively, while those of Pb^2+^, and Ni^2+^ agaist *V. cholerae* J9-62, and N9-4 were 3,200 μg/mL, and 400 μg/mL, respectively. Chen et al. (2021) reported that the maximum MIC values against the *V. cholerae* isolates (*n* = 203) were 800 μg/mL for Cd^2+^, 1600 μg/mL for Zn^2+^, 3200 μg/mL for Pb^2+^, and 1600 μg/mL for Ni^2+^. Similarly, the higher MICs of heavy metals against *V. cholerae* isolates of aquatic animal origins were also reported by Fu et al. (2020). These results suggested possible heavy metal exposure or pollution sources in the aquaculture environments.

Changes in cell biophysical properties have been disclosed as stressors affecting compound and ion transport and cell integrity (Yu P. et al., 2022). Bacterial strains applied different mechanisms to response to heavy metals, such as bond formation between bacterial cell surface and metal ions to decrease their toxicity (Heidari and Panico, 2020; Wróbel et al., 2023). The compositions of the cell surface (e.g., hydroxyl, phosphate, carboxyl, and sulfate) are more involved in linking to metal ions, leading to the deposition of metal ions on the cell surface or the accumulation between the space of the cell membranes (Jarosławiecka and Piotrowska-Seget, 2014; Ashrafi et al., 2022). In this study, our results revealed that the cytoplasma membrane permeability of *V. cholerae* J9-62 and Q6-10 isolates was significantly increased under the Pb^2+^ (200 μg/mL), and Cd^2+^ (12.5 μg/mL) or Zn^2+^ (50 μg/mL) stresses, respectively (*p <* 0.05). The increased inner membrane permeability was also found in *V. parahaemolyticus* N10-8 after treated with a sublethal concentration of Cd^2+^ (50 μg/mL) in our recent study (Yu P. et al., 2022). Likewise, cell membrane fluidity affected most compounds and ions (such as nutrients and heavy metals) to cross the bacterial cytoplasma membrane (Bessa et al., 2018). In this study, we found that the cell membrane fluidity of *V. cholerae* J9-62, and Q6-10 isolates was significantly decreased under the Pb^2+^, and Cd^2+^ or Zn^2+^ stresses, respectively (*p <* 0.05). Hu et al. (2019) reported that the plasma membrane fluidity of *E. coli* and *Phanerochaete chrysosporium* decreased gradually with the increased concentrations of Cd^2+^ (0–80 nM). Most recently, Yu S. et al. (2022) found that the cell membrane fluidity of *V. parahaemolyticus* N10-18 was significantly decreased after treated with the 50 μg/mL of Cd^2+^. Bacteria can adjust membrane lipid composition to control membrane homeostasis in response to environmental changes (Bessa et al., 2018). In this study, the proteome-level analysis provided certain evidence for the results by the biochemical assays. For example, the comparative proteomic analysis revealed that the expression of fatty acid biosynthesis-associated proteins was significantly induced in *V. cholerae* J9-62 under the Pb^2+^ stress (*p <* 0.05). The altered abundance of lipid-metabolism-related proteins likely led to the reduced cell membrane fluidity (Li D. et al., 2022). In addition, cell membrane hydrophobicity affected the activation free energy of passive lipid transport (Rogers et al., 2021). In this study, we observed that the cell membrane hydrophobicity of *V. cholerae* J9-62, and Q6-10 isolates was significantly increased under the Pb^2+^, and Cd^2+^ or Zn^2+^ stresses (*p <* 0.05). Similar case was also found in *V. parahaemolyticus* N10-8 after treated with the Cd^2+^ (50 μg/mL) in our recent study (Yu P. et al., 2022). Exceptionally, the Ni^2+^ (50 μg/mL) stress only increased the cell membrane fluidity of *V. cholerae* N9-4 (*p <* 0.05). Taken, our results, coupled with the previous studies, suggested that the changes in cell biophysical properties were likely additional strategies for *V. cholerae* to survive under the heavy metal stresses.

Consistent with the changes in the bacterial cell biophysical properties, comparative secretomic and proteomic analyses revealed differential extracellular and intracellular proteins in *V. cholerae* J9-62, Q6-10, and N9-4 isolates elicited by the Pb^2+^, Cd^2+^, Zn^2+^, or Ni^2+^ stresses, respectively. Interestingly, the Pb^2+^, Zn^2+^, and Ni^2+^ stresses increased extracellular protein secretion in *V. cholerae* J9-62, Q6-10, and N9-4 isolates (*n* = 5–10), respectively, whereas under the Cd^2+^ stress, *V. cholerae* Q6-10 secreted less extracellular proteins (*n* = 5) than those in the control group. For example, the secretion of enolase (spot A-11) was induced in *V. cholerae* J9-62 under the Pb^2+^ stress. Ling et al. reported that enolase was secreted by alkaliphilic bacterium *Bacillus lehensis* G1 in the alkaline pH condition (Ling et al., 2018). Recently, Zhao et al. (2023) reported that enolase, a Cd resistance-related protein from hyperaccumulator plant *Phytolacca americana,* increased the tolerance of *E. coli* to Cd stress. These results, coupled with our finding in this study, provided evidence for enolase serving as a stress protein under different environmental stresses. In addition, seven to ten metabolic pathways in *V. cholerae* J9-62, Q6-10, and N9-4 isolates were significantly altered under the Pb^2+^, Cd^2+^, Zn^2+^, or Ni^2+^ stresses, respectively. For example, many DIPs were enriched in the starch and sucrose, fructose and mannose, glycolysis and gluconeogenesis, amino acid sugar and nucleotide sugar metabolic pathways in *V. cholerae* J9-62, and Q6-10 under the Pb^2+^, and Cd^2+^ stresses, respectively. Notably, all these pathways were associated with glucose metabolism, the coordination of which may have provided critical material fluxes and energy for cellular activity, especially under the heavy metal stress. Additionally, a close link between the purine/pyrimidine metabolism and antimicrobial stress has been suggested (Sung et al., 2022). In this study, our comparative proteomic data provided the first evidence for such metabolism-related proteins involved in the Zn^2+^ stress in *V. cholerae* Q6-10.

An effective regulation of metal ion homeostasis is essential for bacterial survival in any environment. An eminent mechanism for such homeostasis is ABC transporters (Mandal et al., 2019). In this study, many proteins involved in ABC transporters were produced in the *V. cholerae* isolates under the heavy metal stresses. For example, an ABC transporter substrate-binding protein, an arginine ABC transporter, a sulfate ABC transporter substrate-binding protein, a peptide/nickel transport system substrate-binding protein, and a cysteine/glutathione ABC transporter permease were produced in *V. cholerae* N9-4 under the Ni^2+^ stress. In this study, we found that efflux pumps-associated proteins were produced in the *V. cholerae* isolates under the heavy metal stresses. For example, an efflux transporter outer membrane subunit, and a Co/Zn/Cd efflux system membrane fusion protein were expressed in *V. cholerae* J9-62 under the Pb^2+^ stress; a multidrug efflux RND transporter permease subunit VexB, and a vibriobactin export RND transporter periplasmic adaptor subunit VexG were produced in *V. cholerae* Q6-10 under the Cd^2+^ stress; a multidrug efflux RND transporter permease subunit VexB, and a multidrug efflux RND transporter periplasmic adaptor subunit VexA were expressed in *V. cholerae* Q6-10 under the Zn^2+^ stress. In addition, the expression of iron carriers in *V. cholerae* J9-62, and Q6-10 isolates were induced under the Pb^2+^, and Zn^2+^ stresses, respectively. Most recently, we also found the greatly enhanced expression of Zn/Cd/Hg/Pb-transportation and efflux, and ABC transporters in *V. parahaemolyticus* N10-18 under the Cd stress (Yu P. et al., 2022).

Thiamine was involved in various abiotic stress response in microorganisms, such as drought, high salt, and oxidative stress (Li Y. et al., 2022). In this study, thiamine biosynthesis-associated proteins were produced in *V. cholerae* Q6-10 under the Zn^2+^ stress. The expression of glutathione peroxidase and glutathione reductase were also induced in *V. cholerae* Q6-10 under the Cd^2+^ stress. Fang et al. reported that *C. nantongensis* X1^T^ strain could reduce the cytotoxicity of Cd^2+^ and improve resistance to Cd^2+^ by regulating glutathione metabolism and reducing oxidative stress (Fang et al., 2022). Moreover, in this study, *V. cholerae* Q6-10 was found to significantly enrich the taurine and hypotaurine metabolism under the Cd^2+^ stress. Taurine has been proven to have clear alleviating effects on the damage caused by Cd, Mn, and Pb (Duan et al., 2023).

In this study, we found that the expression of extracellular polysaccharides was induced in *V. cholerae* N9-4, and Q6-10 isolates under the Ni^2+^, and Cd^2+^ stresses, respectively, and that a high-affinity zinc uptake system protein ZnuA (Spot B-13) was secreted by *V. cholerae* Q6-10 under the Zn^2+^ stress, suggesting possible extracellular sequestration of the heavy metals. Overall, our data revealed common defensive strategies developed by the *V. cholerae* isolates under different heavy metal (Pb^2+^, Cd^2+^, Zn^2+^, and Ni^2+^) stresses. On the other hand, different strategies were also observed in the *V. cholerae* isolates to cope with different heavy metal stresses.

Notably, a number of putative virulence-associated proteins were differently produced (*n* = 108) and secreted (*n* = 4) in the *V. cholerae* isolates under the heavy metal stresses. For example, the expression of T6SS-related proteins in *V. cholerae* Q6-10, and N9-4 was induced under the Cd^2+^, and Ni^2+^ stresses, respectively, which are closely associated with the virulence of *V. cholerae* (Crisan and Hammer, 2020). Fang et al. reported that Cd^2+^ (20 mg/L) elicited differential expression of 1,157 genes in *C. nantongensis* X1^T^, including the T6SS-related genes. In addition, in this study, some putative resistance-associated proteins were also differently produced in the *V. cholerae* isolates under the heavy metal stresses. These data suggested an increased health risk of the *V. cholerae* isolates in aquatic products triggered by the heavy metal stresses.

Although the 2D-GE is a powerful technique to study protein alternations in bacteria under environmental stresses, due to the technique limitations of protein spot separation on the 2D-GE gels, not all of the differential extracellular proteins could be identified from the *V. cholerae* isolates under the heavy metal stresses. Similar case for the proteomics data, particularly to the proteins with weaker abundance or at lower expression levels. Additionally, *V. cholerae* is usually challenged by multiple heavy metals in aquatic environments. Therefore, it will be interesting to investigate synergistic effects of different heavy metals on the *V. cholerae* survival in the future research.

## Conclusion

The *V. cholerae* J9-62, Q6-10, and N9-4 isolates of aquatic animal origins showed different heavy metal tolerant profiles. The sublethal concentrations of the Pb^2+^ (200 μg/mL), Cd^2+^ (12.5 μg/mL) and Zn^2+^ (50 μg/mL) stresses at 37°C for 2 h decreased cell membrane fluidity of *V. cholerae* J9-62, and Q6-10 (*p <* 0.05), but increased the bacterial cell surface hydrophobicity and inner membrane permeability (*p <* 0.05). Exceptionally, the Ni^2+^ (50 μg/mL) stress only increased the cell membrane fluidity of *V. cholerae* N9-4 (*p <* 0.05).

The comparative secretomic analysis revealed that *V. cholerae* J9-62, Q6-10, and N9-4 isolates secreted 32 differential proteins under the Cd^2+^ (12.5 μg/mL), Pb^2+^ (200 μg/mL), Ni^2+^ (50 μg/mL), or Zn^2+^ (50 μg/mL) stresses. Meanwhile, a number of DIPs were also identified in the *V. cholerae* isolates, which significantly altered seven to eleven metabolic pathways under the Pb^2+^, Cd^2+^, Zn^2+^, or Ni^2+^ stresses. The comparative secretomic and proteomic analyses revealed common defensive strategies developed by the *V. cholerae* isolates to ameliorate cytotoxicity of the heavy metal (Pb^2+^, Cd^2+^, Zn^2+^, and Ni^2+^) stresses, such as the activation of efflux pump RND transporters, ABC transporters and metal chelators for transportation and effluxing; the expression of glutathione peroxidase for reducing oxidative stress damage; the biosynthesis of EPS for extracellular biosorption and sequestration; and the activation of energy metabolism-related pathways. In addition, different strategies were also observed in the *V. cholerae* isolates to cope with different heavy metal stresses: *V. cholerae* J9-62 reduced the Pb^2+^ cytotoxicity by inducing the biosynthesis of hydrophobic amino acids; *V. cholerae* Q6-10 mitigated the Cd^2+^ damage by inducing taurine accumulation; *V. cholerae* Q6-10 reduced the Zn^2+^ hazard by eliciting the iron–sulfur protein expression and thiamin biosynthesis; and *V. cholerae* N9-4 reduced the Ni^2+^ cytotoxicity by triggering the expression of T6SS-associated proteins.

Remarkably, a number of putative virulence and resistance-associated proteins were differently produced and/or secreted in the *V. cholerae* isolates under the heavy metal stresses, suggested an increased health risk of the *V. cholerae* isolates in aquatic products under the heavy metal conditions.

Overall, the results of this study fill prior gaps of *V. cholerae* in response to the heavy metal stresses, and facilitate better understanding of pathogenesis and MDR resistance of the common waterborne pathogen worldwide.

## Data availability statement

The raw LC-MS/MS data have been deposited in the ProteomeXchange Consortium via the PRoteomics IDEntifications (PRIDE) database (iPRIDE) partner respiratory under the accession number PXD046079.

## Author contributions

BZ: Data curation, Formal analysis, Investigation, Writing – original draft. JX: Data curation, Formal analysis, Writing – original draft. MS: Investigation, Writing – original draft. PY: Writing – original draft. YM: Data curation, Formal analysis, Writing – original draft. LX: Supervision, Writing – review & editing. LC: Conceptualization, Funding acquisition, Project administration, Supervision, Writing – review & editing.
