# Supplementary material for: Comparative secretomic and proteomic analysis reveal multiple defensive strategies developed by Vibrio cholerae against the heavy metal (Cd2+, Ni2+, Pb2+, and Zn2+) stresses
Source: Front Microbiol. 2023 Oct 26;14:1294177. doi: 10.3389/fmicb.2023.1294177 (PMC10637575; doi:10.3389/fmicb.2023.1294177)
Supplement: Supplementary file 1 [file Data_Sheet_1.doc]

**Supplementary Tables**

**Table S1** Phenotypes and toxin genotypes of the *V. cholerae* isolates used in this study.

| ***V. cholerae* strain** | **Origin** | **Toxin genotype** | | | | **Resistance phenotype** | | **Genome (GenBank accession No.)** |
| --- | --- | --- | --- | --- | --- | --- | --- | --- |
|  |  | ***rtxBCD*** | ***tlh*** | ***hlyA*** | ***hapA*** | **Heavy metal** | **Antibiotic** |  |
| J9-62 | *Carassius auratus* | **+** | **+** | **+** | **+** | Pb^2+^/Cr^3+^ | STR | SRR15508183 |
| Q6-10 | *Ctenopharyngodon idellμs* | **+** | − | **+** | **+** | Zn^2+^/Cd^2+^ | AMP/STR | SRR15508376 |
| N9-4 | *Saxidomus. purpuratus* | **+** | **+** | **+** | **+** | Ni^2+^/Zn^2+^ | AMP | *− |

Note: AMP, ampicillin; STR, streptomycin; Cd^2+^, CdCl_2_; Cr^3+^, CrCl_3_; Pb^2+^, PbCl_2_; Zn^2+^, ZnCl_2_; *−, not available.

**Table S2** The MICs and fatality rates of the heavy metals against the *V. cholerae* isolates.

| ***V. cholerae* strain** | **Heavy metal** | **MIC**  **(μg/mL)** | **Sublethal concentration (μg/mL)** | **Fatality rate***  **(%)** |
| --- | --- | --- | --- | --- |
| J9-62 | Pb^2+^ | 3200 | 200 | 42.75 |
| Q6-10 | Cd^2+^ | 400 | 12.5 | 27.98 |
|  | Zn^2+^ | 800 | 50 | 29.7 |
| N9-4 | Ni^2+^ | 400 | 50 | 34.42 |

Note: *, fatality rates of the *V. cholerae* isolates after treated with sublethal concentrations of the heavy metals for 2 h.

**Table S3** Identification of the differential protein spots on the secretomic profiles of the *V. cholerae* isolates under the heavy metal stresses by the LC-MS/MS analysis.

| **Protein spot No.** | **Uniprot No.** | **Protein** | **Gene** | **Sequence coverage (%)** | **MW (Da)** | **PI** | **Putative function** | ***V. cholerae* isolate** | **Heavy metal stress** |
| --- | --- | --- | --- | --- | --- | --- | --- | --- | --- |
| A-1 | A0A655V753 | RbmA protein | *ERS013206_00478* | 5.35 | 19,201.49 | 5.04 | -* | J9-62 | Pb^2+^ |
| A-2 | A0A395TVM7 | Porin_4 domain-containing protein | *BC353_02920* | 10.85 | 36,721.71 | 4.52 | Porin activity, cell outer membrane | J9-62 | Pb^2+^ |
| A-3 | A0A0X1KVQ6 | Uncharacterized protein | *VchoM_00377* | 4.98 | 33,281.89 | 5.54 | -* | J9-62 | Pb^2+^ |
| A-4 | A0A5C2AUZ1 | DUF91 domain-containing protein | *F0315_01105* | 2.42 | 38,056.47 | 4.84 | Nucleic acid binding | J9-62 | Pb^2+^ |
| A-5 | A0A0X1L3V5 | UDP-N-acetylglucosamine 1-carboxyvinyltransferase | *murA* | 16.71 | 44,685.92 | 5.46 | Cell wall formation, adds enolpyruvyl to UDP-N-acetylglucosamine. | J9-62 | Pb^2+^ |
| A-6 | A0A0H3AGK4 | Peptidase, M20A family | *VC0395_A0959* | 15.49 | 39,399.36 | 5.11 | Aminopeptidase activity, metal ion binding | J9-62 | Pb^2+^ |
| A-7 | A0A5C2B2D4 | Glycosyl hydrolase family 26 | *F0315_00990* | 2.67 | 30,029.45 | 5.48 | -* | J9-62 | Pb^2+^ |
| A-8 | A0A5C9T247 | GntP family permease | *FXE67_03300* | 1.75 | 47,298.30 | 6.89 | Gluconate transmembrane transporter activity, integral component of membrane | J9-62 | Pb^2+^ |
| A-9 | A0A5C9Q2V3 | Hydroxyacylglutathione hydrolase | *gloB* | 5.95 | 28,315.81 | 5.3 | Thiolesterase that catalyzes the hydrolysis of S-D-lactoyl-glutathione to form glutathione and D-lactic acid. | J9-62 | Pb^2+^ |
| A-10 | A0A395TTK9 | Thiol: disulfide interchange protein | *BC353_12020* | 4.50 | 22,521.73 | 6.9 | -* | J9-62 | Pb^2+^ |
| A-11 | A0A0K9UTM6 | Enolase | *eno* | 24.94 | 45,806.45 | 5.03 | Catalyzes the reversible conversion of 2-phosphoglycerate into phosphoenolpyruvate. | J9-62 | Pb^2+^ |
| A-12 | Q7WSV3 | Leucine aminopeptidase | *lap* | 43.86 | 18,842.69 | 5.49 | Aminopeptidase activity, metalloexopeptidase activity, proteolysis | J9-62 | Pb^2+^ |
| B-1 | A0A7U8WN22 | UPF0246 protein A53_02455 | *A53_02455* | 2.72 | 28,859.57 | 5.83 | -* | Q6-1 | Cd^2+^ |
| B-2 | C3LNJ1 | Peptidoglycan-binding protein CsiV | *VCM66_1811* | 6.77 | 29,508.28 | 5.91 | -* | Q6-10 | Cd^2+^ |
| B-3 | A0A0H3AHQ2 | Thiosulfate ABC transporter, | *cysP* | 23.12 | 36,910.07 | 6.46 | Sulfur compound binding, sulfate transmembrane transport | Q6-10 | Cd^2+^  Zn^2+^ |
| B-4 | A0A0X1L2I2 | Immunogenic protein | *VchoM_02807* | 24.39 | 35,254.62 | 6.61 | -* | Q6-10 | Cd^2+^ |
| B-5 | A0A544CFY7 | Flagellar hook-length control protein FliK | *FLM02_03925* | 5.56 | 68,547.76 | 4.53 | Bacterial-type flagellum hook, bacterial-type flagellum assembly | Q6-10 | Cd^2+^ |
| B-6 | A0A8G0CCX4 | IutA-like xeno-aerobactin receptor | *iutA* | 1.66 | 79,959.80 | 4.98 | -* | Q6-10 | Cd^2+^  Zn^2+^ |
| B-7 | A0A0K9UXK2 | Putrescine-binding periplasmic protein | *VC274080_021501* | 3.48 | 38,996.08 | 5.15 | Required for the activity of the bacterial periplasmic transport system of putrescine. | Q6-10 | Zn^2+^ |
| B-8 | A0A655ZFS8 | Alanine racemase | *alr* | 7.55 | 23,486.43 | 6.09 | Alanine racemase activity, alanine metabolic process | Q6-10 | Zn^2+^ |
| B-9 | A0A7Z7VJY5 | S8 family peptidase | *EYB64_19945* | 12.64 | 58,185.06 | 6.09 | Serine-type endopeptidase activity, proteolysis, cellular anatomical entity | Q6-10 | Zn^2+^ |
| B-10 | A0A7U8WND8 | Outer membrane protein OmpA | *A53_02322* | 46.42 | 34,285.06 | 5.07 | Porin activity, ion transport, transport | Q6-10 | Zn^2+^ |
| B-11 | A0A7U8WLM2 | AB hydrolase-1 domain-containing protein | *A53_00668* | 3.70 | 29,990.37 | 5.04 | -* | Q6-10 | Zn^2+^ |
| B-12 | A0A0F2TMF9 | ABC transporter substrate-binding protein | *dppA_3* | 12.36 | 57,668.94 | 5.45 | Outer membrane-bounded periplasmic space, transmembrane transport | Q6-10 | Zn^2+^ |
| B-13 | A0A5C2AZI7 | High-affinity zinc uptake system protein ZnuA | *znuA* | 10.10 | 32,952.24 | 5.51 | -* | Q6-10 | Zn^2+^ |
| B-14 | A0A656A6N9 | PrkA serine protein kinase | *ERS013206_03097* | 2.22 | 36,995.17 | 5.51 | Kinase activity, phosphorylation | Q6-10 | Zn^2+^ |
| B-15 | A0A5C2AZ10 | Flagellar hook-associated protein FlgL | *flgL* | 6.30 | 44,958.39 | 5.11 | Structural molecule activity, bacterial-type flagellum-dependent cell motility | Q6-10 | Zn^2+^ |
| C-1 | A0A655YIB7 | Maltodextrin-binding protein | *malE* | 6.57 | 29,821.58 | 4.98 | Periplasmic space, carbohydrate transmembrane transporter activity | N9-4 | Ni^2+^ |
| C-2 | A0A0H3AMP8 | Immunogenic protein | *VC0395_A2848* | 3.35 | 35,254.62 | 6.61 | -* | N9-4 | Ni^2+^ |
| C-3 | A0A395U4X0 | Flagellin | *BC353_00825* | 16.71% | 39,988.84 | 4.78 | Bacterial-type flagellum, extracellular region, structural molecule activity | N9-4 | Ni^2+^ |
| C-4 | A0A655XKD7 | Thiosulfate ABC transporter | *cysP ERS013201_01894* | 3.90 | 36,935.12 | 6.93 | Periplasmic space, sulfur compound binding, sulfate transmembrane transport | N9-4 | Ni^2+^ |
| C-5 | A0A655WKM1 | Small-conductance mechanosensitive channel | *ERS013199_00224* | 10.11 | 10,088.42 | 6.27 | -* | N9-4 | Ni^2+^ |
| -*, not detected. | | | | | | | | | |

**Table S4** Identification of the common protein spots on the secretomic profiles of the *V. cholerae* isolates under the heavy metal stresses by the LC-MS/MS analysis.

| **Protein spot No.** | **Uniprot No.** | **Protein** | **Gene** | **Sequence coverage (%)** | **MW (Da)** | **PI** | **Putative function** | ***V. cholerae* isolate** |
| --- | --- | --- | --- | --- | --- | --- | --- | --- |
| A-a | A0A0H3AHA9 | Oxidoreductase, short-chain dehydrogenase/reductase family | *VC0395_A1193* | 8.73 | 26,791.30 | 5.74 | Oxidoreductase activity | J9-62 |
| A-b | A0A7U8WQZ3 | Transcriptional regulator | *A53_03262* | 14.29 | 28,059.69 | 5.62 | DNA-binding, regulation of DNA-templated transcription, phosphorelay signal transduction system | J9-62 |
| A-c | A0A6G8MV11 | MshD | *mshD* | 7.88 | 21,777.49 | 5.71 | Integral component of membrane | J9-62 |
| A-d | A0A395U307 | Cell filamentation protein Fic | *BC353_18470* | 3.31 | 28,061.22 | 5.4 | -* | J9-62 |
| A-e | A0A6B3LJG0 | Peptidoglycan-binding protein CsiV | *G3T61_08555* | 15.75 | 28,242.91 | 6.32 | -* | J9-62 |
| A-f | A0A8G0CDT3 | 2,3,4,5-tetrahydropyridine-2,6-dicarboxylate N-succinyltransferase | *dapD* | 26.53 | 35,676.32 | 5.99 | 2,3,4,5-tetrahydropyridine-2,6-dicarboxylate N-succinyltransferase activity, magnesium ion binding | J9-62 |
| A-g | A0A5C9T165 | Porin | *FXE67_04795* | 15.70 | 37,043.24 | 4.54 | Cell outer membrane, porin activity | J9-62 |
| A-h | A0A0K9UVU9 | D-serine deaminase transcriptional activator | *VC274080_023463* | 2.55 | 35,885.28 | 6.56 | DNA-binding, Transcription, Transcription regulation | J9-62 |
| A-i | A0A0X1L2R9 | Fructose-bisphosphate aldolase | *VchoM_02858* | 31.01 | 38,918.37 | 4.91 | Fructose-bisphosphate aldolase activity, zinc ion binding, glycolytic process | J9-62 |
| A-j | A0A655Y136 | Flagellin | *flaD_2* | 34.22 | 39,933.75 | 4.89 | Bacterial-type flagellum, extracellular region, structural molecule activity | J9-62 |
| A-k | A5F4Y1 | Chaperonin GroEL 2 | *groEL2* | 42.28 | 57,210.86 | 4.76 | ATP binding, ATP-dependent protein folding chaperone, isomerase activity, unfolded protein binding, protein refolding | J9-62 |
| A-l | C3LTA5 | Chaperone protein DnaK | *dnaK* | 32.60 | 68,760.03 | 4.8 | ATP-dependent protein folding chaperone, unfolded protein binding, ATP binding | J9-62 |
| A-m | A0A0X1KV31 | Glucose-specific PTS system component | *VchoM_00241* | 21.89 | 18,022.46 | 4.56 | Transferase activity, phosphoenolpyruvate-dependent sugar phosphotransferase system | J9-62 |
| A-n | A0A8G0CAM8 | 2,3-bisphosphoglycerate-independent phosphoglycerate mutase | *gpmM (gpmI pgmI)* | 11.96 | 55,398.11 | 4.96 | 2,3-bisphosphoglycerate-independent phosphoglycerate mutase activity, manganese ion binding | J9-62 |
| A-o | A5F6X1 | Trigger factor (TF) | *tig* | 23.33 | 47,953.37 | 5 | Chaperone, isomerase, Rotamase, cell cycle, cell division, protein folding, protein transport | J9-62 |
| A-p | P0C6Q3 | Phosphoglycerate kinase | *pgk* | 18.09 | 40,978.63 | 4.91 | ADP binding, ATP binding, phosphoglycerate kinase activity, gluconeogenesis | J9-62 |
| B-a | A0A5Q6PNB3 | Porin OmpU | *ompU* | 20.56 | 38,499.70 | 4.46 | Porin activity, cell outer membrane | Q6-10 |
| B-b | A0ZPL7 | PBPb domain-containing protein |  | 5.81 | 29,497.54 | 5.8 | -* | Q6-10 |
| B-c | D7HCK1 | Transcriptional regulator | *VCRC385_03063* | 8.98 | 28,059.69 | 5.62 | DNA binding | Q6-10 |
| B-d | A0A085SHN6 | Amino acid ABC transporter substrate-binding protein | *peb1A* | 27.49 | 36,850.41 | 5.19 | -* | Q6-10 |
| B-e | A0A5C2B0M9 | Putrescine-binding periplasmic protein | *F0315_12605* | 3.48 | 38,995.10 | 5.23 | Polyamine binding polyamine transport | Q6-10 |
| B-f | A0A085T4X6 | 2,3-bisphosphoglycerate-independent phosphoglycerate mutase | *gpmM* | 2.16 | 55,356.08 | 5 | Catalyzes the interconversion of 2-phosphoglycerate and 3-phosphoglycerate. | Q6-10 |
| B-g | A0A5C2AUZ1 | DUF91 domain-containing protein | *F0315_01105* | 2.42 | 38,056.47 | 4.84 | Nucleic acid binding | Q6-10 |
| B-h | Q9KV37 | Elongation factor Tu-A (EF-Tu-A) | *tufA* | 22.59 | 43,184.91 | 5.09 | GTP binding, function GTPase activity, function translation elongation factor activity | Q6-10 |
| C-a | A0A7U8WSL5 | Alkyl hydroperoxide reductase C | *A53_00882* | 5.80 | 22,861.80 | 5.37 | -* | N9-4 |
| C-b | A0A0K9V0F0 | Hcp protein | *VC274080_022944* | 16.28 | 19,059.15 | 5.28 | -* | N9-4 |
| C-C | A0A395U7T2 | Elongation factor Ts (EF-Ts) | *tsf BC353_01210* | 2.86 | 29,877.92 | 5.16 | Cytoplasm, translation elongation factor activity, protein biosynthesis | N9-4 |
| C-d | A0A068FR99 | OmpU | *ompU* | 6.25 | 36,089.91 | 4.37 | Cell outer membrane, porin activity | N9-4 |
| C-e | D7HFK4 | DNA-directed RNA polymerase subunit alpha (RNAP subunit alpha) | *rpoA* | 2.42 | 36,415.09 | 4.83 | DNA-directed RNA polymerase complex，DNA binding, DNA-directed 5'-3' RNA polymerase activity, protein dimerization activity, DNA-templated transcription | N9-4 |
| E-f | A0A068FR95 | OmpU | *ompU* | 6.09 | 36,858.65 | 4.44 | Cell outer membrane, porin activity | N9-4 |

*-, not detected.

**Table S5** Identification of putative virulence-associated proteins in secretomes and proteomes of the *V. cholerae* isolates under the heavy metal stresses by the LC-MS/MS analysis.

| **Protein spot No.** | **Uniprot No.** | **Protein** | **Gene** | **Sequence coverage (%)** | **MW (Da)** | **PI** | **Putative function** | ***V. cholerae* isolate** | **Heavy metal stress** | **Reference** |
| --- | --- | --- | --- | --- | --- | --- | --- | --- | --- | --- |
| Putative extracellular virulence-associated proteins | | | | | | | | | | |
| B-10 | A0A7U8WND8 | Outer membrane protein OmpA | *A53_02322* | 46.42 | 34,285.06 | 5.07 | Porin activity, ion transport, transport | Q6-10 | Zn^2+^ | Guan et al., 2021 |
| B-13 | A0A5C2AZI7 | High-affinity zinc uptake system protein ZnuA | *znuA* | 10.10 | 32,952.24 | 5.51 | Involved in the high-affinity zinc uptake transport system | Q6-10 | Zn^2+^ | Ilari et al., 2016 |
| B-14 | A0A656A6N9 | PrkA serine protein kinase | *ERS013206_03097* | 2.22 | 36,995.17 | 5.51 | Kinase activity, phosphorylation | Q6-10 | Zn^2+^ | Kelliher et al., 2021 |
| C-3 | A0A395U4X0 | Flagellin | *BC353_00825* | 16.71% | 39,988.84 | 4.78 | Bacterial-type flagellum, extracellular region, structural molecule activity | N9-4 | Ni^2+^ | Zhao et al., 2022 |
| Putative intracellular virulence-associated proteins | | | | | | | | | | |
| 96 | A0A0K9UP11 | CRP/FNR family transcriptional regulator | *VC274080_022612* | 35.24 | 23,635.19 | 8.4 | DNA binding, DNA-binding transcription factor activity | J9-62 | Pb^2+^ | Goel et al., 2022 |
| 558 | A0A833AFV5 | Type VI secretion system-associated FHA domain protein TagH | *tagH* | 6.87 | 54,873.8 | 4.79 | *- | J9-62 | Pb^2+^ | Wang et al., 2022a |
| 366 | A0A085RV82 | Leucine-responsive regulatory protein | *lrp* | 21.95% | 18789.35 | 7.73 | Sequence-specific DNA binding, regulation of DNA-templated transcription | J9-62 | Pb^2+^ | Schachterle and Sundin, 2019 |
| 980 | A0A2R8EDE0 | Transmembrane regulator ToxS | *toxS* | 4.62 | 19,675.08 | 5.37 | Integral component of membrane | J9-62 | Pb^2+^ | Gubensäk et al., 2021 |
| 1,026 | A0A5C9SQG8 | Octanoyltransferase | *lipB* | 7.76 | 25,078.46 | 6.07 | Lipoyl (octanoyl) transferase activity, cytoplasm, protein lipoylation | J9-62 | Pb^2+^ | Li et al., 2022d |
| 1,192 | Q9KUG3 | Hemolysin, putative | *VC_0558* | 2.82 | 47,406.99 | 6.29 | Flavin adenine dinucleotide binding, plasma membrane, integral component of membrane | J9-62 | Pb^2+^ | Gu et al., 2021 |
| 8 | D7HAU4 | Chaperone clpB | *VCRC385_02463* | 26.93 | 96203.25 | 5.17 | ATP binding, ATP hydrolysis activity | J9-62 | Pb^2+^ | Kędzierska-Mieszkowska and Zolkiewski, 2021 |
| 131 | A0A5C9HJI8 | Outer membrane protein OmpA | *ompA* | 38.01 | 34217.92 | 5 | Cell outer membrane, pore complex, porin activity, monoatomic ion transport | J9-62 | Pb^2+^ | Bunpa et al., 2020 |
| 305 | A0A656A019 | Dihydroxy-acid dehydratase | *ilvD* | 8.73 | 60434.15 | 5.36 | 2 iron, 2 sulfur cluster binding, dihydroxy-acid dehydratase activity, metal ion binding, isoleucine biosynthetic process, valine biosynthetic process | J9-62 | Pb^2+^ | Dutta et al., 2022 |
| 366 | A0A085RV82 | Leucine-responsive regulatory protein | *lrp lrp_2* | 21.95 | 18789.35 | 7.73 | Sequence-specific DNA binding, regulation of DNA-templated transcription | J9-62 | Pb^2+^ | Chen et al., 2019 |
| 430 | A0A8B5ZKT3 | ATP-dependent Clp protease ATP-binding subunit | *FXE67_04435* | 6.13 | 69217.16 | 6.07 | ATP binding, ATP hydrolysis activity, peptidase activity, proteolysis | J9-62 | Pb^2+^ | Lo et al., 2020 |
| 622 | Q9KM65 | CAI-1 autoinducer synthase | *cqsA* | 6.68 | 43593.11 | 5.85 | 8-amino-7-oxononanoate synthase activity, pyridoxal phosphate binding, biotin biosynthetic process, transaminase activity | J9-62 | Pb^2+^ | Gorelik et al., 2019 |
| 795 | Q9KSH7 | Transporter, BCCT family | *VC_1279* | 3.70 | 58464.01 | 5.91 | Plasma membrane, transmembrane transporter activity, nitrogen compound transport | J9-62 | Pb^2+^ | Gregory et al., 2021 |
| 866 | A0A086SL22 | Transcriptional activator HlyU | *BC353_10450* | 14.13 | 10608.09 | 6.58 | *- | J9-62 | Pb^2+^ | Kim, 2020 |
| 871 | A0A0E3W1N3 | Death on curing protein, Doc toxin | *relE2* | 8.00 | 11709.43 | 5.15 | Toxin-antitoxin system | J9-62 | Pb^2+^ | de Castro et al., 2022 |
| 879 | A0A0F2TTM6 | Flagellar basal-body protein | *BC353_00930* | 2.65 | 42600.37 | 5.7 | *- | J9-62 | Pb^2+^ | Nedeljković et al., 2021 |
| 914 | A0A0H3Q4J9 | Transcriptional regulator LuxR family | *VCE_001232* | 4.78 | 22728.05 | 5.2 | DNA binding, phosphorelay signal transduction system, regulation of DNA-templated transcription | J9-62 | Pb^2+^ | Tsevelkhoroloo et al., 2022 |
| 916 | A0A0H3Q4V5 | Putative GTPase | *VCE_000979* | 3.65 | 36327.11 | 5.33 | *- | J9-62 | Pb^2+^ | Bütof et al., 2019 |
| 921 | A0A0H3Q670 | Oxidoreductase short-chain dehydrogenase/reductase family | *VCE_001741* | 3.77 | 25582.05 | 5.58 | Oxidoreductase activity | J9-62 | Pb^2+^ | Dai et al., 2022 |
| 925 | A0A0H4JHH2 | 2-C-methyl-D-erythritol 2,4-cyclodiphosphate synthase | *ispF* | 8.23 | 16837.14 | 5.93 | 2-C-methyl-D-erythritol 2,4-cyclodiphosphate synthase activity, metal ion binding, Process isopentenyl diphosphate biosynthetic process, methylerythritol 4-phosphate pathway, terpenoid biosynthetic process | J9-62 | Pb^2+^ | Mains et al., 2021 |
| 963 | A0A0X1KXW2 | Hemolysin-related protein | *VchoM_01121* | 1.59 | 75329.07 | 5.31 | *- | J9-62 | Pb^2+^ | Sakata et al., 2018 |
| 980 | A0A2R8EDE0 | Transmembrane regulator ToxS | *toxS* | 4.62 | 19675.08 | 5.37 | Membrane | J9-62 | Pb^2+^ | Gubensäk et al., 2021 |
| 997 | A0A544JTY6 | ABC-F family ATPase | *FLM02_02795* | 1.89 | 59920.33 | 5.06 | ATP binding | J9-62 | Pb^2+^ | Fostier et al., 2021 |
| 1,042 | A0A655UGG2 | Histidinol dehydrogenase | *hisD_2* | 4.08 | 29926.91 | 9.01 | Histidinol dehydrogenase activity, metal ion binding, NAD binding | J9-62 | Pb^2+^ | Xu et al., 2022 |
| 1,073 | A0A6A9HGH1 | Cardiolipin synthase A | *cls clsA* | 2.07 | 55280.69 | 6.68 | Plasma membrane, cardiolipin synthase activity, cardiolipin biosynthetic process | J9-62 | Pb^2+^ | Li et al., 2022b |
| 98 | A0A0F2TMF9 | ABC transporter substrate-binding protein | *dppA_3* | 19.88 | 57668.94 | 5.45 | ATP-binding cassette (ABC) transporter complex, outer membrane-bounded periplasmic space, transmembrane transport | Q6-10 | Cd^2+^ | Akhtar and Turner, 2022 |
| 247 | P0C6C6 | Flagellin D | *flaD* | 21.75 | 39903.72 | 4.89 | Bacterial-type flagellum, extracellular region, structural molecule activity | Q6-10 | Cd^2+^ | McGee et al., 1996 |
| 256 | A0A0K9UVQ4 | ParB family chromosome partitioning protein | *VC274080_023250* | 18.27 | 35922.85 | 6.99 | DNA binding | Q6-10 | Cd^2+^ | Socea et al., 2021 |
| 410 | A0A0K9UYR1 | Zinc protease | *VC274080_021767* | 5.10 | 104249.07 | 5.09 | Metal ion binding, metalloendopeptidase activity, proteolysis | Q6-10 | Cd^2+^ | Saleh et al., 2019 |
| 883 | A0A085QLN3 | Porin family protein | *BC353_14625* | 5.00 | 17002.9 | 4.78 | *- | Q6-10 | Cd^2+^ | Diaz et al., 2020 |
| 262 | A0A655XQP5 | Carboxy-terminal protease | *prc* | 7.96 | 73,794.45 | 5.24 | Serine-type endopeptidase activity, proteolysis | Q6-10 | Cd^2+^ | Roy et al., 2020 |
| 593 | B2CKP3 | Hemagglutinin/protease regulatory protein | *hapR* | 13.79 | 23,692.13 | 6.22 | DNA binding, peptidase activity, proteolysis | Q6-10 | Cd^2+^ | Cruite et al., 2018 |
| 349 | U3TIW2 | Cholix toxin | *chxA* | 8.12 | 69,964.08 | 5.04 | NAD^+^-diphthamide ADP-ribosyltransferase activity | Q6-10 | Cd^2+^ | Yahiro et al., 2019 |
| 606 | H9L4Q3 | Hcp protein | *VC_1415 VC_A0017* | 26.74 | 19,059.15 | 5.28 | *- | Q6-10 | Cd^2+^ | Ling et al., 2022 |
| 848 | A5F6G4 | Ferric uptake regulation protein | *fur* | 8.67 | 16,941.95 | 5.81 | DNA-binding transcription activator activity, DNA-binding transcription repressor activity | Q6-10 | Cd^2+^ | Liu et al., 2019 |
| 83 | C3LRQ5 | 50S ribosomal protein L2 | *rplB* | 40.88 | 29,824.9 | 10.83 | rRNA binding, structural constituent of ribosome | Q6-10 | Cd^2+^ | Xu et al., 2021 |
| 413 | A0A085SFX6 | Chemotaxis protein CheV | *D6U24_04905 EYB64_08470* | 14.94 | 34,278 | 5.33 | Chemotaxis, phosphorelay signal transduction system | Q6-10 | Zn^2+^ | Xu et al., 2020a |
| 1,381 | A0A8G0CBM7 | Transcriptional regulator LeuO | *leuO* | 4.39 | 36,188.79 | 5.59 | DNA-binding transcription factor activity | Q6-10 | Zn^2+^ | Sánchez-Popoca et al., 2022 |
| 927 | A0A2K9REL6 | Cholix toxin | *chxA* | 3.35 | 73,215.18 | 5.16 | NAD+-diphthamide ADP-ribosyltransferase activity | Q6-10 | Zn^2+^ | Yahiro et al., 2019 |
| 1,117 | A0A085S070 | Iron-binding protein IscA | *iscA* | 10.28 | 11,717.05 | 4.81 | 2 iron 2 sulfur cluster binding, iron-sulfur cluster assembly | Q6-10 | Zn^2+^ | Li et al., 2019a |
| 695 | A0A0K9UPB7 | Peroxiredoxin | *VC274080_022703* | 8.64 | 26843.2 | 4.93 | Thioredoxin peroxidase activity, cellular response to oxidative stress | Q6-10 | Cd^2+^ | Rocha et al., 2021 |
| 739 | A0A5C9Q2E3 | Cell envelope integrity protein TolA | *tolA* | 5.62 | 40041.89 | 9.16 | Membrane, toxin transmembrane transporter activity, bacteriocin transport | Q6-10 | Cd^2+^ | Li et al., 2022a |
| 750 | A0A5R8KZ68 | MipA/OmpV family protein | *ompV* | 7.39 | 28110.74 | 5.25 | Cell outer membrane | Q6-10 | Cd^2+^ | Xu et al., 2020b |
| 810 | D7HC29 | Isopentenyl-diphosphate delta-isomerase | *VCRC385_02898* | 3.64 | 61026.13 | 5.82 | *- | Q6-10 | Cd^2+^ | Añorga et al., 2020 |
| 819 | D7HGI7 | TonB system receptor | *VCRC385_00227* | 3.24 | 75721.66 | 5.5 | Cell outer membrane, siderophore-iron transmembrane transporter activity, signaling receptor activity | Q6-10 | Cd^2+^ | Chen et al., 2022d |
| 890 | A0A085RXI8 | TetR family transcriptional regulator | *BC353_087* | 5.23 | 17845.24 | 6.23 | DNA binding | Q6-10 | Cd^2+^ | Teper et al., 2019 |
| 894 | A0A085SWV0 | RNA-binding protein | *yhbY* | 9.18 | 11037.87 | 9.52 | RNA binding | Q6-10 | Cd^2+^ | Westermann et al., 2019 |
| 908 | A0A0F0BDL3 | MarR family transcriptional regulator | *mgrA* | 6.92 | 17996.4 | 6.38 | DNA-binding transcription factor activity | Q6-10 | Cd^2+^ | Beggs et al., 2020 |
| 942 | A0A0H3Q134 | Glutamate synthase | *VCE_003522* | 2.15 | 47666.38 | 6.51 | Membrane | Q6-10 | Cd^2+^ | Zhou et al., 2018 |
| 943 | A0A0H3Q1A3 | Phosphate transport regulator | *VCE_003717* | 5.26 | 26076.16 | 5.5 | *- | Q6-10 | Cd^2+^ | Choi et al., 2019 |
| 955 | A0A0H3Q9G7 | Thioredoxin 2 | *VCE_001453* | 6.25 | 15968.1 | 6.7 | Protein-disulfide reductase (NAD(P)) activity | Q6-10 | Cd^2+^ | May et al., 2019 |
| 960 | A0A0H6QYH5 | Polysaccharide deacetylase | *xynD* | 2.20 | 67107.5 | 8.42 | Hydrolase activity, acting on carbon-nitrogen (but not peptide) bonds, carbohydrate metabolic process | Q6-10 | Cd^2+^ | Gao et al., 2019 |
| 1,073 | A0A655XLL7 | Sensor histidine kinase | *rstB_2* | 6.06 | 14743.85 | 6.11 | Protein histidine kinase activity | Q6-10 | Cd^2+^ | Li et al., 2019b |
| 1,183 | D7HCZ4 | ATP-binding protein | *VCRC385_03206* | 1.73 | 50286.61 | 7.56 | *- | Q6-10 | Cd^2+^ | Zhang et al., 2017 |
| 1,217 | Q9KS53 | ATP-dependent RNA helicase RhlE | *VC_1407* | 2.02 | 44201.53 | 8.98 | Cytosol, ATP binding, hydrolase activity, nucleic acid binding, RNA helicase activity, ribosomal large subunit assembly | Q6-10 | Cd^2+^ | Hausmann et al., 2021 |
| 349 | A0A5C2AWI6 | Mannose-1-phosphate guanylyltransferase | *F0315_04155* | 11.25 | 52902.29 | 5.62 | *- | Q6-10 | Zn^2+^ | Taj et al., 2022 |
| 351 | A0A5C9SRP0 | Saccharopine dehydrogenase family protein | *FXE67_18865* | 18.12 | 45957.05 | 5.17 | Oxidoreductase activity | Q6-10 | Zn^2+^ | Taj et al., 2022 |
| 423 | A0A0H3PZM4 | Regulatory protein CysB | *VCE_003174* | 14.20 | 36172.24 | 7.25 | *- | Q6-10 | Zn^2+^ | Chen et al., 2022b |
| 488 | A0A5B0H5G6 | Maltoporin LamB | *lamB* | 9.80 | 43248.99 | 4.52 | Pore complex, porin activity, carbohydrate transmembrane transport | Q6-10 | Zn^2+^ | Islam et al., 2022 |
| 854 | A0A085SSR5 | Adenylate cyclase | *D6U24_03780* | 4.55 | 58691.03 | 5.62 | Adenylate cyclase activity, inorganic triphosphate phosphatase activity | Q6-10 | Zn^2+^ | Ahmad and Sebo, 2020 |
| 956 | A0A5C9GDJ1 | Mannose-6-phosphate isomerase | *manA* | 4.21 | 45010.65 | 5.09 | *- | Q6-10 | Zn^2+^ | Li et al., 2020 |
| 1,183 | A0A0H3Q4R4 | ParE toxin protein | *VCE_000809* | 10.48 | 12158.82 | 9.35 | *- | Q6-10 | Zn^2+^ | Snead et al., 2022 |
| 1,206 | A0A0K9UPW6 | Dihydrofolate reductase | *VC274080_020562* | 4.24 | 18524.17 | 5.7 | Dihydrofolate reductase activity, NADP binding, glycine biosynthetic process, one-carbon metabolic process, tetrahydrofolate biosynthetic process | Q6-10 | Zn^2+^ | He et al., 2020 |
| 1,209 | A0A0K9URW3 | GTPase | *VC274080_023719* | 3.65 | 36327.11 | 5.33 | *- | Q6-10 | Zn^2+^ | Crane et al., 2021 |
| 1,213 | A0A0K9UV04 | LacI family transcriptional regulator | *VC274080_023132* | 2.69 | 37090.32 | 7.62 | DNA binding, regulation of DNA-templated transcription | Q6-10 | Zn^2+^ | Sause et al., 2019 |
| 1,214 | A0A0K9UVA0 | 1,4-alpha-glucan branching enzyme | *VC274080_023182* | 7.07 | 11554.01 | 5.4 | *- | Q6-10 | Zn^2+^ | Posada-Reyes et al., 2022 |
| 1,277 | A0A5B1C2B4 | ArsC family reductase | *F0M16_13410* | 10.69 | 15092.22 | 9.2 | *- | Q6-10 | Zn^2+^ | Chauhan et al., 2019 |
| 1,278 | A0A5B1C3T0 | Transcriptional regulator GcvA | *F0H40_03855* | 3.92 | 34525.04 | 5.94 | DNA-binding transcription factor activity, DNA binding | Q6-10 | Zn^2+^ | Lv et al., 2022 |
| 1,282 | A0A5C2AWD7 | Glycosyltransferase | *F0315_04160* | 2.02 | 44314.75 | 7.6 | *- | Q6-10 | Zn^2+^ | Xu et al., 2023 |
| 1,301 | A0A5Q6PNN7 | AraC family transcriptional regulator | *F0M16_03015* | 2.97 | 33928.69 | 8.8 | DNA-binding transcription factor activity, sequence-specific DNA binding | Q6-10 | Zn^2+^ | Sahebi et al., 2022 |
| 1,307 | A0A655T856 | Asparaginase | *ansA_2* | 4.85 | 22218.47 | 6.9 | Asparaginase activity | Q6-10 | Zn^2+^ | Vimal et al., 2021 |
| 1,308 | A0A655U2G4 | Superoxide dismutase, Mn | *sodA* | 6.36 | 12520.84 | 4.48 | Metal ion binding,  superoxide dismutase activity | Q6-10 | Zn^2+^ | Culbertson et al., 2020 |
| 1,340 | A0A7U8WN73 | Multidrug DMT transporter permease | *A53_02427* | 7.84 | 11128.47 | 4.41 | *- | Q6-10 | Zn^2+^ | Kim et al., 2021 |
| 1,320 | A0A655YV09 | Phosphopantetheine adenylyltransferase | *coaD* | 11.11 | 10982.54 | 6.27 | Pantetheine-phosphate adenylyltransferase activity, coenzyme A biosynthetic process | Q6-10 | Zn^2+^ | Gupta et al., 2021 |
| 1,325 | A0A656AJQ5 | Chemotaxis motA protein | *motA* | 3.04 | 26657.3 | 5.15 | Plasma membrane, bacterial-type flagellum-dependent swarming motility,  chemotaxis | Q6-10 | Zn^2+^ | Hayashi et al., 2018 |
| 1,373 | A0A8B5V9R7 | Restriction endonuclease subunit S | *FLM12_07635* | 2.04 | 48953.95 | 9.01 | DNA binding, endonuclease activity, DNA restriction-modification system | Q6-10 | Zn^2+^ | Kakuta et al., 2020 |
| 1,379 | A0A8G0C9L0 | Cysteine/glutathione ABC transporter ATP-binding protein/permease CydC | *cydC* | 1.57 | 63516.15 | 8.7 | *- | Q6-10 | Zn^2+^ | Poole et al., 2019 |
| 1,404 | C3LTF5 | ABC transporter, permease protein | *VCM66_0863* | 4.89 | 23762.2 | 9.15 | Plasma membrane, transmembrane transport | Q6-10 | Zn^2+^ | Denic et al., 2021 |
| 1,416 | D7H7I2 | Thioredoxin domain-containing protein | *VCRC385_00660* | 2.94 | 34671.09 | 4.94 | *- | Q6-10 | Zn^2+^ | Zhou et al., 2020 |
| 1,420 | D7H9C4 | Trypsin domain-containing protein | *VCRC385_01199* | 1.87 | 58287.94 | 6.17 | Serine-type endopeptidase activity, proteolysis | Q6-10 | Zn^2+^ | Alshammari et al., 2022 |
| 1,460 | Q9KS17 | Histidine kinase | *VC_1444* | 3.70 | 44387.74 | 4.9 | *- | Q6-10 | Zn^2+^ | Cai et al., 2021 |
| 1,472 | Q9KV79 | Transcriptional regulator, MerR family | *VC_0277* | 6.77 | 14916.01 | 5.49 | DNA binding, DNA-binding transcription factor activity, zinc ion binding | Q6-10 | Zn^2+^ | Jiang et al., 2018 |
| 91 | A0A0H3AHZ0 | Acetolactate synthase, catabolic | *alsS* | 27.5 | 61910.3 | 6.19 | Acetolactate synthase activity, magnesium ion binding, thiamine pyrophosphate binding, butanediol metabolic process, carboxylic acid metabolic process | Q6-10 | Zn^2+^ | Tang et al., 2021 |
| 99 | Q9KQ30 | 5'-nucleotidase | *nutA* | 23.51 | 60902.37 | 5.51 | Cell outer membrane, componentouter membrane-bounded periplasmic space, 5'-nucleotidase activity, metal ion binding, nucleotide binding, UDP-sugar diphosphatase activity, XMP 5'-nucleosidase activity, nucleotide catabolic process | Q6-10 | Zn^2+^ | Abouelkhair et al., 2020 |
| 275 | A0A085SHN6 | Amino acid ABC transporter substrate-binding protein | *peb1A* | 25.73 | 36850.41 | 5.19 | *- | Q6-10 | Zn^2+^ | Tian et al., 2018 |
| 104 | A0A0H3AJV5 | Outer membrane protein TolC | *tolC* | 34.70% | 47794.56 | 5.04 | Outer membrane, efflux transmembrane transporter activity | Q6-10 | Zn^2+^ | Pattanayak et al., 2021 |
| 140 | O34242 | Chaperone protein DnaJ | *dnaJ* | 32.28% | 40821.65 | 8.2 | ATP binding, heat shock protein binding, unfolded protein binding, zinc ion binding, chaperone cofactor-dependent protein refolding, DNA replication, protein refolding | Q6-10 | Zn^2+^ | Zhang et al., 2023 |
| 1,047 | Q9K3D4 | RelB protein | *VC_A0349* | 14.75% | 13943.7 | 6.91 | *- | Q6-10 | Zn^2+^ | Eluard et al., 2022 |
| 1,325 | A0A656AJQ5 | Chemotaxis motA protein | *motA* | 3.04% | 26657.3 | 5.15 | Plasma membrane, bacterial-type flagellum-dependent swarming motility, chemotaxis | Q6-10 | Zn^2+^ | Han et al., 2023 |
| 658 | A0A085PIX0 | Reactive intermediate/imine deaminase | *BC353_07035* | 31.78 | 13687.55 | 5.22 | *- | N9-4 | Ni^2+^ | Shang et al., 2021 |
| 758 | A0A8B5VFJ1 | Glycogen debranching protein GlgX | *glgX* | 8.23 | 74994.42 | 6.08 | Glycogen debranching enzyme activity, glycogen catabolic process | N9-4 | Ni^2+^ | Han et al., 2022 |
| 1,218 | D7HDX2 | D-alanyl-D-alanine carboxypeptidase | *VCRC385_03324* | 5.54 | 54368.64 | 6.38 | Serine-type carboxypeptidase activity, proteolysis | N9-4 | Ni^2+^ | Kijek et al., 2019 |
| 1,454 | A0A5C9SY04 | AraC family transcriptional regulator | *FXE67_09145* | 4.46 | 23268.78 | 8.96 | *- | N9-4 | Ni^2+^ | Marghani et al., 2021 |
| 1,497 | A0A655YHB1 | Copper-sensing two-component system response regulator CusR | *ERS013201_02483* | 5.47 | 27797.82 | 5.27 | *- | N9-4 | Ni^2+^ | Novoa-Aponte et al., 2020 |
| 1,573 | C3LQZ3 | MazG protein | *mazG* | 2.88% | 32086.78 | 4.94 | *- | N9-4 | Ni^2+^ | Nigo et al., 2023 |
| 1,046 | A0A086SL22 | Transcriptional activator HlyU | *BC353_10450* | 25.00 | 10608.09 | 6.58 | *- | N9-4 | Ni^2+^ | Kim, 2020 |
| 1,079 | A0A0H3Q9W2 | Transcriptional regulator LuxT | *VCE_001609* | 13.66 | 18749.32 | 6.24 | DNA binding | N9-4 | Ni^2+^ | Eickhoff et al., 2022 |
| 1,083 | A0A0H6G991 | Cholera toxin transcriptional activator | *toxR* | 9.86 | 32505.49 | 5.36 | DNA binding, regulation of DNA-templated transcription | N9-4 | Ni^2+^ | Raskin et al., 2020 |
| 1,131 | A0A5C2B375 | Thioredoxin TrxC | *trxC* | 12.50 | 15967.12 | 7.56 | Protein-disulfide reductase activity | N9-4 | Ni^2+^ | He et al., 2023 |
| 727 | A0A0K9URG6 | LysM domain-containing protein | *VC274080_023764* | 31.91 | 21647.56 | 9.98 | Peptidoglycan binding | N9-4 | Ni^2+^ | Harishchandra et al., 2020 |
| 475 | A0A0H3QD31 | NADPH: quinone reductase | *VCE_000549* | 26.10 | 33782.68 | 4.99 | Oxidoreductase activity | N9-4 | Ni^2+^ | Shukla et al., 2020 |
| 634 | K7S2N6 | Chemotaxis protein CheV | *F0M16_00395* | 24.51 | 34653.78 | 5.82 | Phosphorelay signal transduction system | N9-4 | Ni^2+^ | Du et al., 2018 |
| 810 | A0A086SQN7 | OmpA family protein | *oprF* | 16.51 | 23465.93 | 4.82 | Membrane, calcium ion binding | N9-4 | Ni^2+^ | Paulsson et al., 2021 |
| 729 | A0A0K9UYB0 | NADH-dependent flavin oxidoreductase | *VC274080_023354* | 12.10% | 38301.76 | 5.03 | FMN binding, oxidoreductase activity | N9-4 | Ni^2+^ | Xie et al., 2021 |
| 1,386 | A0A0X1KWD4 | 2-methylisocitrate lyase (2-MIC) (MICL) | *prpB* | 3.25% | 33736.33 | 5.61 | Magnesium ion binding, methylisocitrate lyase activity, propionate catabolic process, 2-methylcitrate cycle | N9-4 | Ni^2+^ | Yan et al., 2019 |
| 1,011 | A0A833A8X5 | Type II secretion system protein | *F0H40_10735* | 13.84% | 16057.94 | 4.82 | Membrane | N9-4 | Ni^2+^ | Ingel et al., 2023 |
| 812 | A0A0H3AHF5 | Outer membrane protein OmpV | *ompV* | 13.23% | 28110.74 | 5.25 | Cell outer membrane | N9-4 | Ni^2+^ | Kaur et al., 2021 |
| 572 | D7H7U2 | Oligopeptide ABC transporter, ATP-binding protein | VCRC385_00742 | 23.46% | 36094.68 | 6.04 | ATP binding, ATP hydrolysis activity, peptide transport | N9-4 | Ni^2+^ | Poole et al., 2019 |

*-, not detected.

**Table S6** Identification of putative resistance-associated proteins in secretomes and proteomes of the *V. cholerae* isolates under the heavy metal stresses by the LC-MS/MS analysis.

| **Protein spot No.** | **Uniprot No.** | **Protein** | **Gene** | **Sequence coverage (%)** | **MW (Da)** | **PI** | **Putative function** | ***V. cholerae* isolate** | **Heavy metal stress** | **Reference** |
| --- | --- | --- | --- | --- | --- | --- | --- | --- | --- | --- |
| Putative extracellular resistance-associated proteins | | | | | | | | | | |
| B-9 | A0A7Z7VJY5 | S8 family peptidase | *EYB64_19945* | 12.64 | 58,185.06 | 6.09 | Serine-type endopeptidase activity, proteolysis, cellular anatomical entity | Q6-10 | Zn^2+^ | Zhang et al., 2022 |
| Putative intracellular resistance-associated proteins | | | | | | | | | | |
| 82 | C3LMN9 | Catalase-peroxidase (CP) | *katG* | 26.8 | 80,649.92 | 5.79 | Catalase activity, heme binding, metal ion binding, hydrogen peroxide catabolic process | Q6-10 | Cd^2+^ | Jiang et al., 2022 |
| 799 | C3LR65 | Transcriptional reguator, TetR family | *VCM66_0317* | 8.68 | 28,374.48 | 7.19 | DNA binding | Q6-10 | Cd^2+^ | Colclough et al., 2019 |
| 119 | A0A0H3AH83 | Alkyl hydroperoxide reductase C | *VC0395_A0264* | 50.24 | 22,861.8 | 5.37 | Peroxiredoxin activity | Q6-10 | Cd^2+^ | Park et al., 2022 |
| 563 | A0A395TSF2 | Multidrug transporter AcrB | *BC353_03110* | 4.43 | 110839.52 | 5.32 | Transmembrane transporter activity, plasma membrane, integral component of membrane | Q6-10 | Zn^2+^ | Yamasaki et al., 2022 |
| 880 | A0A0H3AI79 | Transcriptional reguator, TetR family | *VC0395_A2735* | 8.68 | 28374.48 | 7.19 | DNA binding | Q6-10 | Zn^2+^ | Colclough et al., 2019 |
| 1,133 | A0A0F0AT26 | TetR family transcriptional regulator | *ttgR* | 4.51 | 28604.65 | 6.31 | DNA binding | Q6-10 | Zn^2+^ | Feng et al., 2023 |
| 780 | D7HE56 | Riboflavin synthase | *VCRC385_01524* | 11.06 | 23559.72 | 5.27 | *- | J9-62 | Pb^2+^ | Jalal et al., 2022 |
| 858 | A0A085T698 | DNA starvation/stationary phase protection protein | *BC353_11025* | 8.97 | 17,873.07 | 5.15 | DNA binding, ferric iron binding, oxidoreductase activity, acting on metal ions | J9-62 | Pb^2+^ | Tseng et al., 2019 |
| 997 | A0A544JTY6 | ABC-F family ATPase | *FLM02_02795* | 1.89 | 59,920.33 | 5.06 | ATP binding | J9-62 | Pb^2+^ | Fostier et al., 2021 |
| 1,141 | C3LVK1 | Putative glutathione S-transferase | *VCM66_A0544* | 5.42 | 23,433.47 | 5.98 | Glutathione transferase activity | J9-62 | Pb^2+^ | Gao et al., 2020 |
| $940 | A0A0H7EH35 | Small ribosomal subunit biogenesis GTPase RsgA | rsgA | 2.55 | 39150.92 | 5.91 | Hydrolase, RNA-binding, Ribosome biogenesis | J9-62 | Pb^2+^ | Rocchio et al., 2019 |
| 1,126 | A0A8G0FV30 | L,D-transpeptidase family protein | *KTC41_13325* | 2.29 | 34123.16 | 7.72 | *- | J9-62 | Pb^2+^ | Zandi et al., 2019 |
| 1,155 | D7HD65 | Lipoprotein signal peptidase | *lspA* | 4.68 | 19419.35 | 7.86 | Plasma membrane, aspartic-type endopeptidase activity, proteolysis | J9-62 | Pb^2+^ | Garland et al., 2020 |
| 1,028 | A0A5C9SVE0 | Efflux transporter outer membrane subunit | *FXE67_13680* | 1.73 | 50602.97 | 5.39 | Cell outer membrane, efflux transmembrane transporter activity | J9-62 | Pb^2+^ | Mitra et al., 2021 |
| 950 | A0A0K9UVH7 | Glutathione peroxidase | *VC274080_023321* | 12.20 | 9426.66 | 8.62 | *- | J9-62 | Pb^2+^ | Chen et al., 2022c |
| 922 | A0A0H3Q6M9 | Iron-sulfur cluster assembly protein CyaY | *cyaY* | 9.62 | 11860.04 | 4.25 | Cytoplasm, ferric iron binding, iron-sulfur cluster assembly | J9-62 | Pb^2+^ | Saha et al., 2016 |
| 424 | A0A0H3Q008 | Leucine-responsive regulatory protein | *VCE_003171* | 28.66 | 18789.35 | 7.73 | Sequence-specific DNA binding, regulation of DNA-templated transcription | Q6-10 | Zn^2+^ | Ziegler and Freddolino, 2021 |
| 945 | A0A544E6Q6 | 5'-methylthioadenosine/S-adenosylhomocysteine nucleosidase | *mtnN* | 9.52 | 24510.89 | 4.77 | Methylthioadenosine nucleosidase activity, L-methionine salvage from methylthioadenosine, L-methionine salvage from S-adenosylmethionine | Q6-10 | Zn^2+^ | Cornell et al., 2020 |
| 920 | A0A0X1L0H4 | Acetyl-CoA synthetase | *VchoM_02133* | 3.64 | 73824.4 | 5.59 | Acetoacetate-CoA ligase activity, lipid metabolic process | Q6-10 | Zn^2+^ | Liu et al., 2022 |
| 1,307 | A0A655T856 | Asparaginase | *ansA_2* | 4.85 | 22218.47 | 6.9 | Asparaginase activity | Q6-10 | Zn^2+^ | Vimal et al., 2021 |
| 1,317 | A0A655Y8B7 | ABC transporter, ATP-binding protein YrbF | *mlaF* | 15.49 | 7808.79 | 4.37 | ATP binding, hydrolase activity | Q6-10 | Zn^2+^ | Su et al., 2018 |
| 1,328 | A0A656AS69 | ABC transporter ATPase | *ERS013200_00812* | 2.85 | 48024.82 | 5.18 | ATP binding, ATP hydrolysis activity, DNA binding | Q6-10 | Zn^2+^ | Zheng et al., 2018 |
| 1,351 | A0A7U8WRD0 | ABC transporter | *A53_01713* | 6.44 | 25361.74 | 9.1 | *- | Q6-10 | Zn^2+^ | Greene et al., 2018 |
| 1,327 | A0A656AK91 | Thiamine monophosphate kinase | *thiL* | 5.24 | 22836.74 | 5.44 | Thiamine-phosphate kinase activity, phosphorylation, thiamine biosynthetic process | Q6-10 | Zn^2+^ | Kim et al., 2020 |
| 1,336 | A0A7U8WLX7 | Coenzyme A biosynthesis bifunctional protein CoaBC | *coaBC* | 3.26 | 42644.87 | 6.56 | *- | Q6-10 | Zn^2+^ | Domingo et al., 2019 |
| 1,340 | A0A7U8WN73 | Multidrug DMT transporter permease | *A53_02427* | 7.84 | 11128.47 | 4.41 | *- | Q6-10 | Zn^2+^ | Kim et al., 2021 |
| 1,416 | D7H7I2 | Thioredoxin domain-containing protein | *VCRC385_00660* | 2.94 | 34671.09 | 4.94 | *- | Q6-10 | Zn^2+^ | Zhou et al., 2020 |
| 1,341 | A0A7U8WNK6 | Nucleotide-binding protein | *A53_02632* | 4.20 | 32502.82 | 6.19 | *- | Q6-10 | Zn^2+^ | Daniel et al., 2018 |
| 1,379 | A0A8G0C9L0 | Cysteine/glutathione ABC transporter ATP-binding protein/permease CydC | *cydC* | 1.57 | 63516.15 | 8.7 | *- | Q6-10 | Zn^2+^ | Poole et al., 2019 |
| 1,406 | C3LTT4 | Iron-sulfur cluster carrier protein | *mrp* | 2.36% | 40716.52 | 5.99 | ATP binding, ATP hydrolysis activity, ATP-dependent FeS chaperone activity, iron-sulfur cluster binding, metal ion binding | Q6-10 | Zn^2+^ | Huang et al., 2019 |
| 269 | A0A8G0CCA7 | Multidrug efflux RND transporter permease subunit VexB | *vexB* | 6.85% | 111808 | 5.26 | *- | Q6-10 | Zn^2+^ | Yu et al., 2020 |
| 1,080 | Q9KSH8 | Transcriptional regulator, MarR family | *VC_1278* | 8.18% | 17996.4 | 6.38 | DNA-binding transcription factor activity, regulation of DNA-templated transcription | Q6-10 | Zn^2+^ | Beggs et al., 2020 |
| 1,178 | D7HBB7 | Glutathione peroxidase | *VCRC385_02636* | 9.80% | 11827.42 | 8.98 | *- | Q6-10 | Cd^2+^ | Fang et al., 2023 |
| 466 | Q9KTG5 | Exopolysaccharide biosynthesis protein, putative | *VC_0937* | 5.97 | 83017.74 | 6.27 | Plasma membrane, ATP binding, protein tyrosine kinase activity, extracellular polysaccharide biosynthetic process, lipopolysaccharide biosynthetic process, phosphorylation | Q6-10 | Cd^2+^ | Heidari and Panico, 2020 |
| 403 | A0A0K9UML9 | Glutathione hydrolase proenzyme | *VC274080_020320* | 6.34 | 62627.17 | 5.82 | Glutathione hydrolase activity, hypoglycin A gamma-glutamyl transpeptidase activity, leukotriene C4 gamma-glutamyl transferase activity, glutathione biosynthetic process, glutathione catabolic process | Q6-10 | Cd^2+^ | Wang et al., 2022b |
| 1,201 | C3LPG5 | Bacterioferritin comigratory protein | *bcp* | 18.71 | 17135.36 | 6.18 | Antioxidant activity | N9-4 | Ni^2+^ | Gupta et al., 2022 |
| 1,335 | A0A0H3Q161 | Transport ATP-binding protein CydC | *VCE_002511* | 1.57 | 63485.08 | 8.63 | Membrane, ATP hydrolysis activity, glutathione transmembrane transport | N9-4 | Ni^2+^ | Poole et al., 2019 |
| 1,372 | A0A0K9UWD1 | Universal stress protein family 1 | *VC274080_023109* | 2.56 | 35328.3 | 5.92 | *- | N9-4 | Ni^2+^ | Cui et al., 2021 |
| 1,055 | A0A0F2TW44 | AI-2E family transporter | *ydiK* | 8.86 | 40046.97 | 6.44 | Membrane | N9-4 | Ni^2+^ | Li et al., 2022c |
| 1,134 | A0A5C9SU05 | NAD(P)H:quinone oxidoreductase | *wrbA* | 12.90 | 20123.72 | 6.49 | NAD(P)H dehydrogenase (quinone) activity | N9-4 | Ni^2+^ | Lee et al., 2021 |
| 1,501 | A0A655ZB43 | L, D-transpeptidase YcbB | *ERS013200_01930* | 2.68 | 42498.92 | 5.61 | Transferase activity, peptidoglycan biosynthetic process | N9-4 | Ni^2+^ | Atze et al., 2022 |
| 901 | A0A395UF36 | SAM-dependent methyltransferase | *BC353_04685* | 16.23 | 21619.31 | 4.9 | Methyltransferase activity, methylation | N9-4 | Ni^2+^ | G and Singh, 2022 |
| 1,058 | A0A0H3AGC7 | Putative glutathione S-transferase | *VC0395_0529* | 13.79 | 23433.47 | 5.98 | Transferase activity | N9-4 | Ni^2+^ | Tao et al., 2022 |
| 398 | A0A5C9Q9U0 | SDR family oxidoreductase | *FXE67_10090* | 34.55 | 26726.23 | 5.39 | Cytoplasm, acetoacetyl-CoA reductase activity, poly-hydroxybutyrate biosynthetic process | N9-4 | Ni^2+^ | Li et al., 2021 |
| 522 | Q9KKV4 | ABC transporter, ATP-binding protein | *VC_A0996* | 13.41 | 68690.93 | 5.96 | Membrane, ATP binding, transmembrane transport | N9-4 | Ni^2+^ | Amawi et al., 2019 |
| 796 | A0A395U3Q1 | Histidine kinase | *BC353_00200* | 47.54 | 13848.06 | 5.44 | Kinase activity, phosphorylation | N9-4 | Ni^2+^ | Chen et al., 2022a |
| 122 | A0A7Z7YG93 | Type VI secretion system contractile sheath large subunit | *tssC* | 36.38 | 55592.41 | 5.42 | *- | N9-4 | Ni^2+^ | Stietz et al., 2019 |
| 543 | A0A085RY02 | Hcp family type VI secretion system effector (Hemolysin co-regulated protein) | *hcpA* | 34.88 | 19086.22 | 5.46 | *- | N9-4 | Ni^2+^ | Fei et al., 2022 |
| 1,394 | A0A0X1L4R8 | Transposase | *VchoM_03525* | 2.13 | 42236.08 | 6.23 | DNA binding, transposase activity, transposition, DNA-mediated | N9-4 | Ni^2+^ | Spínola-Amilibia et al., 2023 |
| 1,173 | A0A7Z7VLZ7 | Vibriobactin export RND transporter periplasmic adaptor subunit VexG | *vexG* | 5.97 | 38814.21 | 8.58 | Membrane, transmembrane transporter activity, response to chemical | N9-4 | Ni^2+^ | Yu et al., 2020 |
| 1,543 | A0A8B5VEG9 | Cysteine/glutathione ABC transporter permease/ATP-binding protein CydD | *cydD* | 1.34 | 65860.34 | 6.96 | Membrane, ABC-type transporter activity, ATP binding, ATP hydrolysis activity, cysteine transport | N9-4 | Ni^2+^ | Poole et al., 2019 |
| 559 | A0A0H5YVG6 | Sulfate ABC transporter substrate-binding protein | *cysP* | 17.72 | 36920.06 | 6.14 | Periplasmic space, sulfur compound binding, sulfate transmembrane transport | N9-4 | Ni^2+^ | Amawi et al., 2019 |
| 1,191 | A0A8G0CA31 | Exopolysaccharide biosynthesis beta-barrel protein VpsM | *vpsM* | 7.04 | 44961.43 | 4.79 | *- | N9-4 | Ni^2+^ | Raghavan et al., 2023 |
| 1,115 | A0A395TU00 | Lytic murein transglycosylase | *BC353_12530* | 2.93 | 74342.04 | 9.58 | Periplasmic space, hydrolase activity, hydrolyzing O-glycosyl compounds, lytic transglycosylase activity | N9-4 | Ni^2+^ | Liang et al., 2023 |
| 1,132 | A0A5C9QSV8 | Inositol-1-monophosphatase | *suhB* | 13.86 | 29132.01 | 7.01 | Inositol monophosphate 1-phosphatase activity, phosphatidylinositol phosphate biosynthetic process | N9-4 | Ni^2+^ | Wang et al., 2020 |

*-, not detected.

Table S7 Oligonucleotide primers used in the RT-PCR assay.

| Primer | Gene | Sequence (5’ to 3’) | Product length (bp) | Reference |
| --- | --- | --- | --- | --- |
| *Vc_J9_62_murA-F* | *murA* | YCCNGGNTTYCCNACNGAYATG | 142 | This study |
| *Vc_J9_62_murA-R* |  | YTCNGCYTTNGCNCCCAT |  |  |
| *Vc_J9_62_ gloB-F* | *gloB* | SNATHCCNGCNTTYGAYGA | 168 | This study |
| *Vc_J9_62_ gloB-R* |  | CNCCDATRTGRTCRTGRTGRTG |  |  |
| *Vc_J9_62_ rsmA-F* | *rsmA* | GNAAYGAYGTNCAYYTNGGNCA | 158 | This study |
| *Vc_J9_62_ rsmA-R* |  | NGGYTCNGTDATNGCNCC |  |  |
| *Vc_J9_62_ mdtP-F* | *mdtP* | TAYCCNGAYYTNGCNACNATG | 111 | This study |
| *Vc_J9_62_ mdtP-R* |  | YTGRTANCKYTGCCACCA |  |  |
| *Vc_Q6_10_ cysP-F* | *cysP* | SNGCNGCNGAYCARACNAT | 182 | This study |
| *Vc_Q6_10_ cysP-R* |  | RTCNGCNGCNARNCCYTG |  |  |
| *Vc_Q6_10_ mrp-F* | *mrp* | GGGNAARAARGGNGARGTNATG | 208 | This study |
| *Vc_Q6_10_ mrp-R* |  | YTGYTTNGCDATCCARTC |  |  |
| *Vc_Q6_10_ cyaY-F* | *cyaY* | TNGARMGNATHGARGCNGC | 140 | This study |
| *Vc_Q6_10_ cyaY-R* |  | NARCCADATYTCNCKCAT |  |  |
| *Vc_Q6_10_ thiE-F* | *thiE* | GNGTNYTNCCNGARGCNATGA | 188 | This study |
| *Vc_Q6_10_ thiE-F* |  | NGTNGCNARNACCCANGC |  |  |
| *Vc_N9_4_ BC353_00825 -F*  *Vc_N9_4_ BC353_00825 -R* | *BC353_00825* | CNGARGGNGCNATGAAYGA  TGRTANGTYTTNCCNCCC | 298 | This study |
| *Vc_N9_4_gltD-F*  *Vc_N9_4_gltD-R* | *gltD* | CNCARGTNGARATHATGCCNAT  GNGCNGCRTCYTTCCANAC | 179 | This study |
| *16S RNA-F* | *16s RNA* | GACACGGTCCAGACTCCTAC | 179 | Yu et al., 2022 |
| *16S RNA-R* |  | GGTGCTTCTTCTGTCGCTAAC |  |  |

**Supplementary Figure**


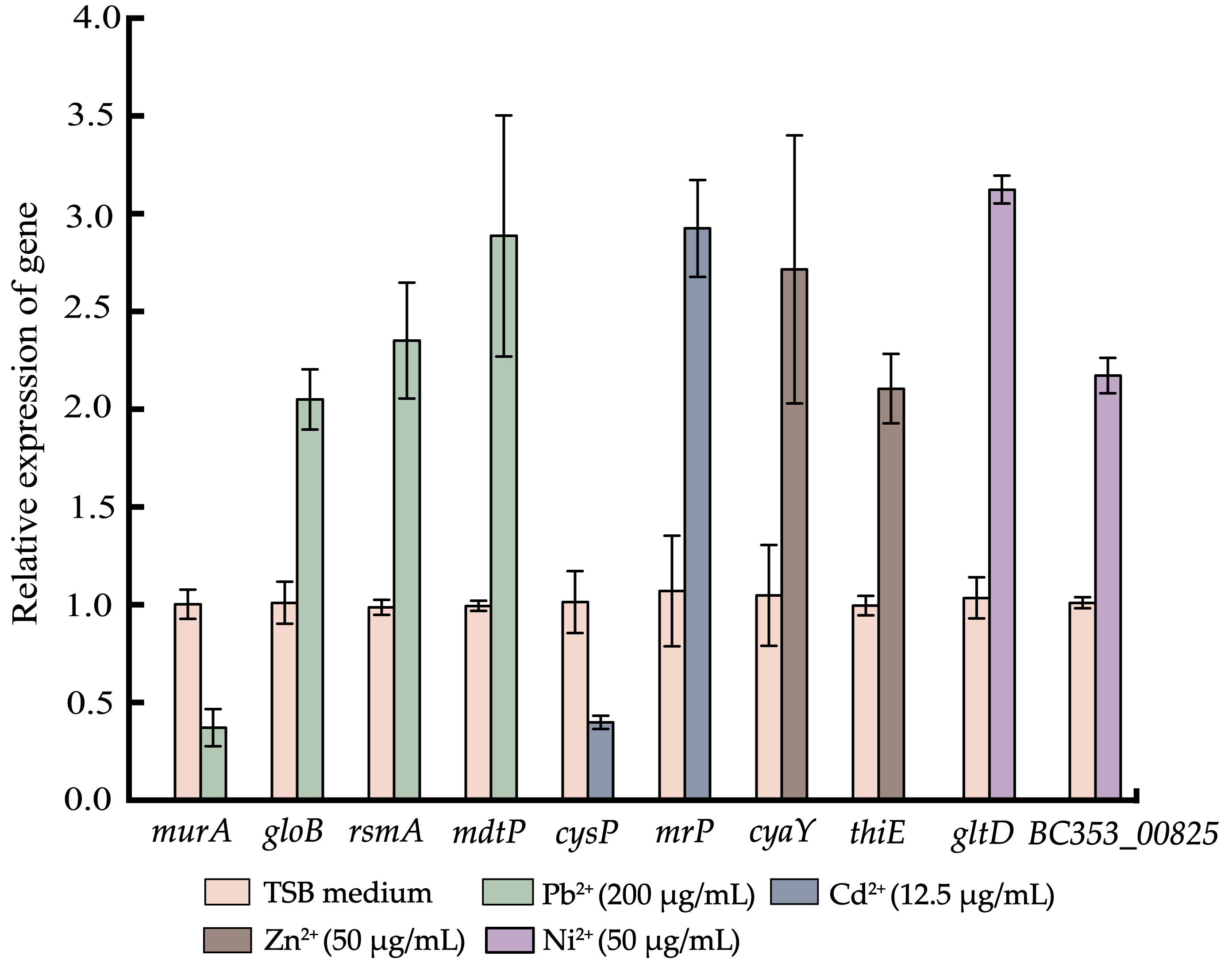


Figure S1 The relative expression of the representative differential proteins by the qRT-PCR assay. *V. cholerae* J9-62 was treated with the Pb^2+^ (200 μg/mL); *V. cholerae* Q6-10 with the Cd^2+^(12.5 μg/mL), or Zn^2+^ (50 μg/mL); and *V. cholerae* N9-4 with Ni (50 μg/mL) for 2 h, respectively.

**References**

Abouelkhair, M. A., Frank, L. A., Bemis, D. A., Giannone, R. J., and Kania, S. A. (2020). *Staphylococcus pseudintermedius* 5'-nucleotidase suppresses canine phagocytic activity. *Vet. Microbiol*. 246, 108720. https://doi./org/10.1016/j.vetmic.2020.108720.

Ahmad, J. N., and Sebo, P. (2020). Adenylate cyclase toxin tinkering with monocyte-macrophage differentiation. *Front. Immunol*. 11, 2181. https://doi./org/10.3389/fimmu.2020.02181.

Akhtar, A. A., and Turner, D. P. (2022). The role of bacterial ATP-binding cassette (ABC) transporters in pathogenesis and virulence: Therapeutic and vaccine potential. *Microb. Pathog*. 171, 105734. https://doi./org/10.1016/j.micpath.2022.105734.

Alshammari, A., Alasmari, A. F., Alharbi, M., Ali, N., Muhseen, Z. T., Ashfaq, U. A., Ud-Din, M., Ullah, A., Arshad, M., and Ahmad, S. (2022). Novel chimeric vaccine candidate development against *Leptotrichia buccalis*. *Int. J. Environ. Res. Public Health*. 19, 10742. https://doi./org/10.3390/ijerph191710742.

Amawi, H., Sim, H. M., Tiwari, A. K., Ambudkar, S. V., and Shukla, S. (2019). ABC transporter-mediated multidrug-resistant cancer. *Adv. Exp. Med. Biol*. 1141, 549–580. https://doi./org/10.1007/978-981-13-7647-4_12.

Añorga, M., Pintado, A., Ramos, C., De Diego, N., Ugena, L., Novák, O., and Murillo, J. (2020). Genes ptz and idi, coding for cytokinin biosynthesis enzymes, are essential for tumorigenesis and in planta growth by *P. syringae* pv. Savastanoi ncppb 3335. *Front. Plant Sci*. 11, 1294. https://doi./org/10.3389/fpls.2020.01294.

Atze, H., Liang, Y., Hugonnet, J. E., Gutierrez, A., Rusconi, F., and Arthur, M. (2022). Heavy isotope labeling and mass spectrometry reveal unexpected remodeling of bacterial cell wall expansion in response to drugs. *Elife.* 11, e72863. https://doi./org/10.7554/eLife.72863.

Beggs, G. A., Brennan, R. G., and Arshad, M. (2020). Marr family proteins are important regulators of clinically relevant antibiotic resistance. *Protein Sci*. 29, 647–653. https://doi./org/10.1002/pro.3769.

Bunpa, S., Chaichana, N., Teng, J. L. L., Lee, H. H., Woo, P. C. Y., Sermwittayawong, D., Sawangjaroen, N., and Sermwittayawong, N. (2020). Outer membrane protein a (OmpA) is a potential virulence factor of *Vibrio alginolyticus* strains isolated from diseased fish. *J. Fish Dis*. 43, 275–284. https://doi./org/10.1111/jfd.13120.

Bütof, L., Große, C., Lilie, H., Herzberg, M., and Nies, D. H. (2019). Interplay between the zur regulon components and metal resistance in *Cupriavidus metallidurans*. *J. Bacteriol*. 201, e00192-19. https://doi./org/10.1128/jb.00192-19.

Cai, E., Sun, S., Deng, Y., Huang, P., Sun, X., Wang, Y., Chang, C., and Jiang, Z. (2021). Histidine kinase Sln1 and Camp/PkA signaling pathways antagonistically regulate *Sporisorium scitamineum* mating and virulence via transcription factor Prf1. *J. Fungi*. 7, 610. https://doi./org/10.3390/jof7080610.

Chauhan, D., Srivastava, P. A., Agnihotri, V., Yennamalli, R. M., and Priyadarshini, R. (2019). Structure and function prediction of arsenate reductase from *Deinococcus indicus* DR1. *J. Mol. Model*. 25, 15. https://doi./org/10.1007/s00894-018-3885-3.

Chen, K. Y., Rathod, J., Chiu, Y. C., Chen, J. W., Tsai, P. J., and Huang, I. H. (2019). The transcriptional regulator Lrp contributes to toxin expression, sporulation, and swimming motility in *Clostridium difficile*. *Front. Cell Infect.* *Microbiol*. 9, 356. https://doi./org/10.3389/fcimb.2019.00356.

Chen, T., Shi, Y., Peng, C., Tang, L., Chen, Y., Wang, T., Wang, Z., Wang, S., and Li, Z. (2022c). Transcriptome analysis on key metabolic pathways in *Rhodotorula mucilaginosa* under Pb(II) stress. *Appl. Environ.* *Microbiol*. 88, e0221521. https://doi./org/10.1128/aem.02215-21.

Chen, Y., Song, K., Chen, X., Li, Y., Lv, R., Zhang, Q., Cui, Y., Bi, Y., Han, Y., Tan, Y., Du, Z., Yang, R., Qi, Z., and Song, Y. (2022d). Attenuation of *Yersinia pestis* fyua mutants caused by iron uptake inhibition and decreased survivability in macrophages. *Front. Cell Infect. Microbiol*. 12, 874773. https://doi./org/10.3389/fcimb.2022.874773.

Chen, H., Yu, C., Wu, H., Li, G., Li, C., Hong, W., Yang, X., Wang, H., and You, X. (2022a). Recent advances in histidine kinase-targeted antimicrobial agents. *Front. Chem*. 10, 866392. https://doi./org/10.3389/fchem.2022.866392.

Chen, M., Zhang, W., Han, L., Ru, X., Cao, Y., Hikichi, Y., Ohnishi, K., Pan, G., and Zhang, Y. (2022b). A cysb regulator positively regulates cysteine synthesis, expression of type III secretion system genes, and pathogenicity in *Ralstonia solanacearum*. *Mol. Plant Pathol*. 23, 679–692. https://doi./org/10.1111/mpp.13189.

Choi, S., Choi, E., Cho, Y. J., Nam, D., Lee, J., and Lee, E. J. (2019). The salmonella virulence protein mgtc promotes phosphate uptake inside macrophages. *Nat. Commun*. 10, 3326. https://doi./org/10.1038/s41467-019-11318-2.

Colclough, A. L., Scadden, J., and Blair, J. M. A. (2019). Tetr-family transcription factors in gram-negative bacteria: Conservation, variation and implications for efflux-mediated antimicrobial resistance. *BMC Genomics*. 20, 731. https://doi./org/10.1186/s12864-019-6075-5.

Cornell, K. A., Knippel, R. J., Cortright, G. R., Fonken, M., Guerrero, C., Hall, A. R., Mitchell, K. A., Thurston, J. H., Erstad, P., Tao, A., Xu, D., and Parveen, N. (2020). Characterization of 5'-methylthioadenosine/s-adenosylhomocysteine nucleosidases from *Borrelia burgdorferi*: Antibiotic targets for lyme disease. *Biochim. Biophys.* *Acta. Gen. Subj*. 1864, 129455. https://doi./org/10.1016/j.bbagen.2019.129455.

Crane, S. D., Banerjee, S. K., Eichelberger, K. R., Kurten, R. C., Goldman, W. E., and Pechous, R. D. (2021). The *Yersinia pestis* gtpase bipa promotes pathogenesis of primary pneumonic plague. *Infect. Immun*. 89, e00673-20. https://doi./org/10.1128/iai.00673-20.

Cruite, J., Succo, P., Raychaudhuri, S., and Kull, F. J. (2018). Crystal structure of an inactive variant of the quorum-sensing master regulator HapR from the protease-deficient non-o1, non-o139 *vibrio cholerae* strain V2. *Acta. Crystallogr. F. Struct. Biol. Commun.* 74(Pt 6), 331–336. https://doi./org/10.1107/s2053230x18006519.

Cui, X., Zhang, P., Hu, Y., Chen, C., Liu, Q., Guan, P., and Zhang, J. (2021). Genome-wide analysis of the universal stress protein a gene family in vitis and expression in response to abiotic stress. *Plant Physiol. Biochem*. 165, 57–70. https://doi./org/10.1016/j.plaphy.2021.04.033.

Culbertson, E. M., Bruno, V. M., Cormack, B. P., and Culotta, V. C. (2020). Expanded role of the cu-sensing transcription factor mac1p in *Candida albicans*. *Mol. Microbiol*. 114, 1006–1018. https://doi./org/10.1111/mmi.14591.

Dai, L., Li, H., Huang, J. W., Hu, Y., He, M., Yang, Y., Min, J., Guo, R. T., and Chen, C. C. (2022). Structure-based rational design of a short-chain dehydrogenase/reductase for improving activity toward mycotoxin patulin. *Int.* *J. Biol. Macromol*. 222(Pt A), 421–428. https://doi./org/10.1016/j.ijbiomac.2022.09.121.

Daniel, J., Abraham, L., Martin, A., Pablo, X., and Reyes, S. (2018). RV2477C is an antibiotic-sensitive manganese-dependent ABC-F ATPase in *Mycobacterium tuberculosis*. *Biochem. Biophys. Res. Commun*. 495, 35–40. https://doi./org/10.1016/j.bbrc.2017.10.168.

De Castro, G. V., Worm, D. J., Grabe, G. J., Rowan, F. C., Haggerty, L., de la Lastra, A. L., Popescu, O., Helaine, S., and Barnard, A. (2022). Characterization of the key determinants of Phd antitoxin mediated doc toxin inactivation in *salmonella*. *ACS Chem. Biol*. 17, 1598–1606. https://doi./org/10.1021/acschembio.2c00276.

Denic, M., Turlin, E., Michel, V., Fischer, F., Khorasani-Motlagh, M., Zamble, D., Vinella, D., and de Reuse, H. (2021). A novel mode of control of nickel uptake by a multifunctional metallochaperone. *PLOS Pathog*. 17, e1009193. https://doi./org/10.1371/journal.ppat.1009193.

Diaz, N., Lico, C., Capodicasa, C., Baschieri, S., Dessì, D., Benvenuto, E., Fiori, P. L., and Rappelli, P. (2020). Production and functional characterization of a recombinant predicted pore-forming protein (tvsaplip12) of *Trichomonas vaginalis* in nicotiana benthamiana plants. *Front. Cell Infect. Microbiol*. 10, 581066. https://doi./org/10.3389/fcimb.2020.581066.

Domingo, R., van der Westhuyzen, R., Hamann, A. R., Mostert, K. J., Barnard, L., Paquet, T., Tjhin, E. T., Saliba, K. J., van Otterlo, W. A. L., and Strauss, E. (2019). Overcoming synthetic challenges in targeting coenzyme a biosynthesis with the antimicrobial natural product CJ-15,801. *Medchemcomm.* 10, 2118–2125. https://doi./org/10.1039/c9md00312f.

Du, X., Kong, K., Tang, H., Tang, H., Jiao, X., and Huang, J. (2018). The novel protein cj0371 inhibits chemotaxis of *Campylobacter jejuni*. *Front. Microbiol*. 9, 1904. https://doi./org/10.3389/fmicb.2018.01904.

Dutta, S., Corsi, I. D., Bier, N., Koehler, T. M. (2022). Brnq-type branched-chain amino acid transporters influence *Bacillus anthracis* growth and virulence. *Mbio*. 13, e0364021. https://doi./org/10.1128/mbio.03640-21.

Eickhoff, M. J., Fei, C., Cong, J. P., and Bassler, B. L. (2022). Luxt is a global regulator of low-cell-density behaviors, including type III secretion, siderophore production, and aerolysin production, in *Vibrio harveyi*. *Mbio*. 13, e0362121. https://doi./org/10.1128/mbio.03621-21.

Eluard, B., Nuan-Aliman, S., Faumont, N., Collares, D., Bordereaux, D., Montagne, A., Martins, I., Cagnard, N., Caly, M., Taoui, O., Lordello, L., Lehmann-Che, J., Tesson, B., Martinez-Climent, J. A., Copie-Bergman, C., Haioun, C., Tilly, H., Bonsang, B., Vincent-Salomon, A., Jais, J. P., Jardin, F., Leroy, K., Maiuri, M. C., Kroemer, G., Molina, T. J., Feuillard, J., and Baud, V. (2022). The alternative Relb NF-κb subunit is a novel critical player in diffuse large B-cell lymphoma. *Blood*. 139, 384–398. https://doi./org/10.1182/blood.2020010039.

Fang, Y. H., Zhang, Y. M., Yue, S. Y., Peng, J. J., Liu, C. X., and Wang, C. H. (2023). Improving catalytic activity, acid-tolerance, and thermal stability of glutathione peroxidase by systematic site-directed selenocysteine incorporation. *Mol. Biotechnol*. Online ahead of print. https://doi./org/10.1007/s12033-023-00682-6.

Fei, N., Ji, W., Yang, L., Yu, C., Qiao, P., Yan, J., Guan, W., Yang, Y., and Zhao, T. (2022). Hcp of the type VI secretion system (T6SS) in *Acidovorax citrulli* group II strain Aac5 has a dual role as a core structural protein and an effector protein in colonization, growth ability, competition, biofilm formation, and ferric iron absorption. *Int. J. Mol. Sci*. 23, 9632. https://doi./org/10.3390/ijms23179632.

Feng, M., Xie, Y., Mao, W., Lu, Y., Wang, Y., Li, H., and Zhang, C. (2023). Efficient biodegradation of Tris-(2-chloroisopropyl) phosphate by a novel strain *Amycolatopsis sp*. Ft-1: Process optimization, mechanism studies and toxicity changes. *J. Hazard. Mater*. 443(Pt A), 130149. https://doi./org/10.1016/j.jhazmat.2022.130149.

Fostier, C. R., Monlezun, L., Ousalem, F., Singh, S., Hunt, J. F., and Boël, G. (2021). ABC-F translation factors: From antibiotic resistance to immune response. *FEBS Lett*. 595, 675–706. https://doi./org/10.1002/1873-3468.13984.

G, G., and Singh, J. (2022). Dithiothreitol causes toxicity in *C. elegans* by modulating the methionine-homocysteine cycle. *Elife*. 11, e76021. https://doi./org/10.7554/eLife.76021.

Gao, J., Chen, B., Lin, H., Liu, Y., Wei, Y., Chen, F., and Li, W. (2020). Identification and characterization of the glutathione S-transferase (GST) family in radish reveals a likely role in anthocyanin biosynthesis and heavy metal stress tolerance. *Gene.* 743, 144484. https://doi./org/10.1016/j.gene.2020.144484.

Gao, F., Zhang, B. S., Zhao, J. H., Huang, J. F., Jia, P. S., Wang, S., Zhang, J., Zhou, J. M., and Guo, H. S. (2019). Deacetylation of chitin oligomers increases virulence in soil-borne fungal pathogens. *Nat. Plants*. 5, 1167–1176. https://doi./org/10.1038/s41477-019-0527-4.

Garland, K., Pantua, H., Braun, M. G., Burdick, D. J., Castanedo, G. M., Chen, Y. C., Cheng, Y. X., Cheong, J., Daniels, B., Deshmukh, G., Fu, Y., Gibbons, P., Gloor, S. L., Hua, R., Labadie, S., Liu, X., Pastor, R., Stivala, C., Xu, M., Xu, Y., Zheng, H., Kapadia, S. B., and Hanan, E. J. (2020). Optimization of globomycin analogs as novel gram-negative antibiotics. *Bioorg. Med. Chem. Lett*. 30, 127419. https://doi./org/10.1016/j.bmcl.2020.127419.

Goel, D., Kumar, S., Joshi, G. K., Rai, P., and Bhatnagar, R. (2022). Crp/Fnr family protein binds to promoters of *atxA* and *sodmn* genes that regulate the expression of exotoxins in *Bacillus anthracis*. *Protein Expr. Purif*. 193, 106059. https://doi./org/10.1016/j.pep.2022.106059.

Gorelik, O., Levy, N., Shaulov, L., Yegodayev, K., Meijler, M. M., and Sal-Man, N. (2019). *Vibrio cholerae* autoinducer-1 enhances the virulence of enteropathogenic *Escherichia coli*. *Sci Rep*. 9, 4122. https://doi./org/10.1038/s41598-019-40859-1.

Gregory, G. J., Boas, K. E., and Boyd, E. F. (2021). The organosulfur compound dimethylsulfoniopropionate (DMSP) is utilized as an osmoprotectant by *Vibrio* species. *Appl. Environ. Microbiol*. 87, e02235-20. https://doi./org/10.1128/aem.02235-20.

Greene, N. P., Kaplan, E., Crow, A., and Koronakis, V. (2018). Corrigendum: Antibiotic resistance mediated by the MacB ABC transporter family: A structural and functional perspective. *Front. Microbiol*. 9, 2318. https://doi./org/10.3389/fmicb.2018.02318.

Gu, H., Cai, X., Zhang, X., Luo, J., Zhang, X., Hu, X., Cai, W., and Li, G. (2021). A previously uncharacterized two-component signaling system in uropathogenic *Escherichia coli* coordinates protection against host-derived oxidative stress with activation of hemolysin-mediated host cell pyroptosis. *PLOS Pathog*. 17, e1010005. https://doi./org/10.1371/journal.ppat.1010005.

Guan, Q., Bhowmick, B., Upadhyay, A., and Han, Q. (2021). Structure and functions of bacterial outer membrane protein a, a potential therapeutic target for bacterial infection. *Curr. Top. Med. Chem*. 21, 1129–1138. https://doi./org/10.2174/1568026621666210705164319.

Gubensäk, N., Wagner, G. E., Schrank, E., Falsone, F. S., Berger, T. M. I., Pavkov-Keller, T., Reidl, J., and Zangger, K. (2021). The periplasmic domains of *Vibrio cholerae* ToxR and ToxS are forming a strong heterodimeric complex independent on the redox state of ToxR cysteines. *Mol. Microbiol*. 115, 1277–1291. https://doi./org/10.1111/mmi.14673.

Gupta, D. N., Dalal, V., Savita, B. K., Alam, M. S., Singh, A., Gubyad, M., Ghosh, D. K., Kumar, P., and Sharma, A. K. (2022). Biochemical characterization and structure-based in silico screening of potent inhibitor molecules against the 1 cys peroxiredoxin of bacterioferritin comigratory protein family from *Candidatus liberibacter* asiaticus. *J. Biomol. Struct. Dyn*. 1–13. https://doi./org/10.1080/07391102.2022.2096118.

Gupta, A., Sharma, P., Singh, T. P., and Sharma, S. (2021). Phosphopantetheine adenylyltransferase: A promising drug target to combat antibiotic resistance. *Biochim. Biophys. Acta. Proteins Proteom*. 1869, 140566. https://doi./org/10.1016/j.bbapap.2020.140566.

Han, A. R., Kim, H., Park, J. T., and Kim, J. W. (2022). Characterization of a cold-adapted debranching enzyme and its role in glycogen metabolism and virulence of *Vibrio vulnificus* mo6-24/o. *J. Microbiol*. 60, 375–386. https://doi./org/10.1007/s12275-022-1507-3.

Han, Q., Wang, S. F., Qian, X. X., Guo, L., Shi, Y. F., He, R., Yuan, J. H., Hou, Y.J., and Li, D. F. (2023). Flagellar brake protein YcgR interacts with motor proteins MotA and Flig to regulate the flagellar rotation speed and direction. *Front. Microbiol*. 14, 1159974. https://doi./org/10.3389/fmicb.2023.1159974.

Ilari, A., Pescatori, L., Di Santo, R., Battistoni, A., Ammendola, S., Falconi, M., Berlutti, F., Valenti, P., and Chiancone, E. (2016). Salmonella enterica serovar typhimurium growth is inhibited by the concomitant binding of Zn(II) and a pyrrolyl-hydroxamate to znua, the soluble component of the znuabc transporter. *Biochim. Biophys. Acta*. 1860, 534–541. https://doi./org/10.1016/j.bbagen.2015.12.006.

Harishchandra, D. L., Zhang, W., Li, X., Chethana, K. W. T., Hyde, K. D., Brooks, S., Yan, J., and Peng, J. (2020). A lysm domain-containing protein ltlysm1 is important for vegetative growth and pathogenesis in woody plant pathogen *Lasiodiplodia theobromae*. *Plant Pathol. J*. 36, 323–334. https://doi./org/10.5423/ppj.Oa.05.2020.0084.

Hausmann, S., Gonzalez, D., Geiser, J., and Valentini, M. (2021). The DEAD-box RNA helicase RhlE2 is a global regulator of *Pseudomonas aeruginosa* lifestyle and pathogenesis. *Nucleic. Acids. Res*. 49, 6925–6940. https://doi./org/10.1093/nar/gkab503.

Hayashi, N., Furue, Y., Kai, D., Yamada, N., Yamamoto, H., Nakano, T., and Oda, M. (2018). Sulfated vizantin suppresses mucin layer penetration dependent on the flagella motility of *Pseudomonas aeruginosa* pao1. *PLOS One.* 13, e0206696. https://doi./org/10.1371/journal.pone.0206696.

He, J., Liu, S., Fang, Q., Gu, H., and Hu, Y. (2023). The thioredoxin system in *Edwardsiella piscicida* contributes to oxidative stress tolerance, motility, and virulence. *Microorganisms*. 11, 827. https://doi./org/10.3390/microorganisms11040827.

He, J., Qiao, W., An, Q., Yang, T., and Luo, Y. (2020). Dihydrofolate reductase inhibitors for use as antimicrobial agents. *Eur. J. Med. Chem*. 195, 112268. https://doi./org/10.1016/j.ejmech.2020.112268.

Heidari, P., and Panico, A. (2020). Sorption mechanism and optimization study for the bioremediation of Pb(II) and Cd(II) contamination by two novel isolated strains Q3 and Q5 of *Bacillus sp.* *Int. J. Environ. Res. Public Health*. 17, 4059. https://doi./org/10.3390/ijerph17114059.

Huang, N., Mao, J., Zhao, Y., Hu, M., and Wang, X. (2019). Multiple transcriptional mechanisms collectively mediate copper resistance in *Cupriavidus gilardii* CR3. *Environ. Sci. Technol*. 53, 4609–4618. https://doi./org/10.1021/acs.est.8b06787.

Ingel, B., Castro, C., Burbank, L., Her, N., De Anda, N. I., Way, H., Wang, P., and Roper, C. (2023). Xylella fastidiosa requires the type II secretion system for pathogenicity and survival in grapevine. *Mol. Plant. Microbe. Interact*. Online ahead of print. https://doi./org/10.1094/mpmi-03-23-0027-r.

Islam, S. I., Mou, M. J., and Sanjida, S. (2022). Application of reverse vaccinology to design a multi-epitope subunit vaccine against a new strain of *Aeromonas veronii*. *J. Genet. Eng. Biotechnol*. 20, 118. https://doi./org/10.1186/s43141-022-00391-8.

Jalal, K., Khan, K., Hayat, A., Ahmad, D., Alotaibi, G., Uddin, R., Mashraqi, M. M., Alzamami, A., Aurongzeb, M., and Basharat, Z. (2022). Mining therapeutic targets from the antibiotic-resistant *Campylobacter coli* and virtual screening of natural product inhibitors against its riboflavin synthase. *Mol. Divers*. 27, 793–810. https://doi./org/10.1007/s11030-022-10455-z.

Jiang, X., Dai, J., Zhang, X., Wu, H., Tong, J., Shi, J., and Fang, W. (2022). Enhanced Cd efflux capacity and physiological stress resistance: The beneficial modulations of *Metarhizium robertsii* on plants under cadmium stress. *J. Hazard. Mater*. 437, 129429. https://doi./org/10.1016/j.jhazmat.2022.129429.

Jiang, X., Li, X., Sun, S., and Jiang, L. (2018). The transcriptional regulator varn contributes to salmonella typhimurium growth in macrophages and virulence in mice. *Res. Microbiol*. 169, 214–221. https://doi./org/10.1016/j.resmic.2018.03.003.

Kakuta, N., Nakano, R., Nakano, A., Suzuki, Y., Tanouchi, A., Masui, T., Horiuchi, S., Endo, S., Kakuta, R., Ono, Y., and Yano, H. (2020). A novel mismatched PCR-restriction fragment length polymorphism assay for rapid detection of GyrA and ParC mutations associated with fluoroquinolone resistance in *Acinetobacter baumannii*. *Ann. Lab. Med*. 40, 27–32. https://doi./org/10.3343/alm.2020.40.1.27.

Kaur, D., Gandhi, S., and Mukhopadhaya, A. (2021). *Salmonella typhimurium* adhesin OmpV activates host immunity to confer protection against systemic and gastrointestinal infection in mice. *Infect. Immun*. 89, e0012121. https://doi./org/10.1128/iai.00121-21.

Kędzierska-Mieszkowska, S., and Zolkiewski, M. (2021). Hsp100 molecular chaperone ClpB and its role in virulence of bacterial pathogens. *Int. J. Mol. Sci*. 22, 5319. https://doi./org/10.3390/ijms22105319.

Kelliher, J. L., Grunenwald, C. M., Abrahams, R. R., Daanen, M. E., Lew, C. I., Rose, W. E., and Sauer, J. D. (2021). Pasta kinase-dependent control of peptidoglycan synthesis via reom is required for cell wall stress responses, cytosolic survival, and virulence in *Listeria monocytogenes*. *PLOS Pathog*. 17, e1009881. https://doi./org/10.1371/journal.ppat.1009881.

Kijek, T. M., Mou, S., Bachert, B. A., Kuehl, K. A., Williams, J. A., Daye, S. P., Worsham, P. L., and Bozue, J. A. (2019). The D-alanyl-d-alanine carboxypeptidase enzyme is essential for virulence in the Schu S4 strain of *francisella tularensis* and a dacD mutant is able to provide protection against a pneumonic challenge. *Microb. Pathog*. 137, 103742. https://doi./org/10.1016/j.micpath.2019.103742.

Kim, B. S. (2020). Spatiotemporal regulation of *Vibrio* exotoxins by Hlyu and other transcriptional regulators. *Toxins*. 12, 544. https://doi./org/10.3390/toxins12090544.

Kim, J., Cater, R. J., Choy, B. C., and Mancia, F. (2021). Structural insights into transporter-mediated drug resistance in infectious diseases. *J. Mol. Biol*. 433(16), 167005. https://doi./org/10.1016/j.jmb.2021.167005.

Kim, H. J., Lee, H., Lee, Y., Choi, I., Ko, Y., Lee, S., and Jang, S. (2020). The Thil enzyme is a valid antibacterial target essential for both thiamine biosynthesis and salvage pathways in *Pseudomonas aeruginosa*. *J. Biol. Chem.* 295, 10081–10091. https://doi./org/10.1074/jbc.RA120.013295.

Lee, W. S., Ham, W., and Kim, J. (2021). Roles of NAD(P)H:Quinone oxidoreductase 1 in diverse diseases. *Life.* 11, 1301. https://doi./org/10.3390/life11121301.

Li, J., Ren, X., Fan, B., Huang, Z., Wang, W., Zhou, H., Lou, Z., Ding, H., Lyu, J., and Tan, G. (2019a). Zinc toxicity and iron-sulfur cluster biogenesis in *Escherichia coli*. *Appl. Environ. Microbiol*. 85(9). https://doi./org/10.1128/aem.01967-18.

Li, X., Fan, X., Shi, Z., Xu, J., Cao, Y., Zhang, T., and Pan, D. (2022c). AI-2E family transporter protein in *Lactobacillus acidophilus* exhibits AI-2 exporter activity and relate with intestinal juice resistance of the strain. *Front. Microbiol*. 13, 908145. https://doi./org/10.3389/fmicb.2022.908145.

Li, X., Gao, X., Yuan, J., Wang, F., Xu, X., Wang, C., Liu, H., Guan, W., Zhang, J., and Xu, G. (2022d). The miR-33a-5p/CRPT axis mediates ovarian cancer cell behaviors and chemoresistance via the regulation of the TGF-β signal pathway. *Front. Endocrinol*. 13, 950345. https://doi./org/10.3389/fendo.2022.950345.

Li, W., Hu, J., Li, L., Zhang, M., Cui, Q., Ma, Y., Su, H., Zhang, X., Xu, H., and Wang, M. (2022b). New mutations in cls lead to daptomycin resistance in a clinical vancomycin- and daptomycin-resistant *Enterococcus faecium* strain. *Front. Microbiol*. 13, 896916. https://doi./org/10.3389/fmicb.2022.896916.

Li, Q., Li, Z., Fei, X., Tian, Y., Zhou, G., Hu, Y., Wang, S., and Shi, H. (2022a). The role of TolA, TolB, and TolR in cell morphology, OMVs production, and virulence of *Salmonella choleraesuis*. *AMB Express*. 12, 5. https://doi./org/10.1186/s13568-022-01347-4.

Li, Z., Liu, H., Bode, A., and Luo, X. (2021). Emerging roles of dehydrogenase/reductase member 2 (dhrs2) in the pathology of disease. *Eur. J. Pharmacol*. 898, 173972. https://doi./org/10.1016/j.ejphar.2021.173972.

Li, Z., Liu, X., Nakanishi, H., and Gao, X. D. (2020). Encapsulation of mannose-6-phosphate isomerase in yeast spores and its application in l-ribose production. *J. Agric. Food Chem*. 68, 6892–6899. https://doi./org/10.1021/acs.jafc.0c02399.

Li, J., Zhu, F., and Li, J. (2019b). Expression of the histidine kinase gene *sshk* correlates with dimethachlone resistance in *Sclerotinia sclerotiorum*. *Phytopathology.* 109, 395–401. https://doi./org/10.1094/phyto-05-18-0156-r.

Liang, Y., Zhao, Y., Kwan, J. M. C., Wang, Y., and Qiao, Y. (2023). *Escherichia coli* has robust regulatory mechanisms against elevated peptidoglycan cleavage by lytic transglycosylases. *J. Biol. Chem*. 299, 104615. https://doi./org/10.1016/j.jbc.2023.104615.

Ling, L., Ren, A., Lu, Y., Zhang, Y., Zhu, H., Tu, P., Li, H., and Chen, D. (2022). The synergistic effect and mechanisms of flavonoids and polysaccharides from *Houttuynia cordata* on H1N1-induced pneumonia in mice. *J. Ethnopharmacol*. 302(Pt A), 115761. https://doi./org/10.1016/j.jep.2022.115761.

Liu, M., Liu, N., Wang, J., Fu, S., Wang, X., and Chen, D. (2022). Acetyl-CoA synthetase 2 as a therapeutic target in tumor metabolism. *Cancers.* 14, 2896. https://doi./org/10.3390/cancers14122896.

Liu, J., Tian, Y., Zhao, Y., Zeng, R., Chen, B., Hu, B., and Walcott, R. R. (2019). Ferric uptake regulator (Fura) is required for *Acidovorax citrulli* virulence on watermelon. *Phytopathology.* 109, 1997–2008. https://doi./org/10.1094/phyto-05-19-0172-r.

Lo, H. H., Liao, C. T., Li, C. E., Chiang, Y. C., and Hsiao, Y. M. (2020). The Clpx gene plays an important role in bacterial attachment, stress tolerance, and virulence in *Xanthomonas campestris* pv. Campestris. *Arch. Microbiol*. 202, 597–607. https://doi./org/10.1007/s00203-019-01772-3.

Lv, J., Zhu, J., Wang, T., Xie, X., Wang, T., Zhu, Z., Chen, L., Zhong, F., and Du, H. (2022). The role of the two-component QseBC signaling system in biofilm formation and virulence of hypervirulent *Klebsiella pneumoniae* ATCC43816. *Front. Microbiol*. 13, 817494. https://doi./org/10.3389/fmicb.2022.817494.

Mains, D. R., Eallonardo, S. J., and Freitag, N. E. (2021). Identification of *Listeria monocytogenes* genes contributing to oxidative stress resistance under conditions relevant to host infection. *Infect. Immun*. 89, e00700-20. https://doi./org/10.1128/iai.00700-20.

Marghani, D., Ma, Z., Centone, A. J., Huang, W., Malik, M., and Bakshi, C. S. (2021). An AraC/XylS family transcriptional regulator modulates the oxidative stress response of *Francisella tularensis*. *J. Bacteriol.* 203, e0018521. https://doi./org/10.1128/jb.00185-21.

May, H. C., Yu, J. J., Zhang, H., Wang, Y., Cap, A. P., Chambers, J. P., Guentzel, M. N., Arulanandam, B. P. (2019). Thioredoxin-a is a virulence factor and mediator of the type IV pilus system in *Acinetobacter baumannii.* *PLOS One*. 14, e0218505. https://doi./org/10.1371/journal.pone.0218505.

McGee, K., Hörstedt, P., and Milton, D. L. (1996). Identification and characterization of additional flagellin genes from *Vibrio anguillarum*. *J. Bacteriol*. 178, 5188–5198. https://doi./org/10.1128/jb.178.17.5188-5198.1996.

Mitra, A., Chatterjee, S., Kataki, S., Rastogi, R. P., and Gupta, D. K. (2021). Bacterial tolerance strategies against lead toxicity and their relevance in bioremediation application. *Environ. Sci. Pollut. Res. Int*. 28, 14271–14284. https://doi./org/10.1007/s11356-021-12583-9.

Nedeljković, M., Sastre, D.E., Sundberg, E.J. (2021). Bacterial flagellar filament: A supramolecular multifunctional nanostructure. *Int. J. Mol. Sci*. 22, 7521. https://doi./org/10.3390/ijms22147521.

Nigo, F., Nakagawa, R., Hirai, Y., Imai, L., Suzuki, Y., Furuta, K., and Kaito, C. (2023). *Staphylococcus aureus* MazG hydrolyzes oxidized guanine nucleotides and contributes to oxidative stress resistance. *Biochimie.* 209, 52–60. https://doi./org/10.1016/j.biochi.2023.02.001.

Novoa-Aponte, L., Xu, C., Soncini, F. C., and Argüello, J. M. (2020). The two-component system copRS maintains subfemtomolar levels of free copper in the periplasm of *Pseudomonas aeruginosa* using a phosphatase-based mechanism. *mSphere.* 5, e01193-20. https://doi./org/10.1128/mSphere.01193-20.

Park, M., Kim, J., Feinstein, J., Lang, K. S., Ryu, S., and Jeon, B. (2022). Development of fluoroquinolone resistance through antibiotic tolerance in *Campylobacter jejuni*. Microbiol. *Spectr*. 10, e0166722. https://doi./org/10.1128/spectrum.01667-22.

Pattanayak, B. S., Dehury, B., Priyadarshinee, M., Jha, S., Beuria, T. K., Soren, D., and Mallick, B. C. (2021). Kanamycin-mediated conformational dynamics of *Escherichia coli* outer membrane protein TolC. *Front. Mol. Biosci*. 8, 636286. https://doi./org/10.3389/fmolb.2021.636286.

Paulsson, M., Kragh, K. N., Su, Y. C., Sandblad, L., Singh, B., Bjarnsholt, T., and Riesbeck, K. (2021). Peptidoglycan-binding anchor is a *Pseudomonas aeruginosa* OmpA family lipoprotein with importance for outer membrane vesicles, biofilms, and the periplasmic shape. *Front. Microbiol*. 12, 639582. https://doi./org/10.3389/fmicb.2021.639582.

Poole, R. K., Cozens, A. G., and Shepherd, M. (2019). The CydDC family of transporters. *Res. Microbiol*. 170, 407–416. https://doi./org/10.1016/j.resmic.2019.06.003.

Posada-Reyes, A. B., Balderas-Martínez, Y. I., Ávila-Ríos, S., Vinuesa, P., and Fonseca-Coronado, S. (2022). An epistatic network describes oppA and glgB as relevant genes for *Mycobacterium tuberculosis*. *Front. Mol. Biosci*. 9, 856212. https://doi./org/10.3389/fmolb.2022.856212.

Raghavan, P. S., Potnis, A. A., Gupta, S., Gadly, T., Kushwah, N., and Rajaram, H. (2023). Interlink between exod (alr2882), exopolysaccharide synthesis and metal tolerance in *Nostoc sp.* strain PCC7120: Insight into its role, paralogs and evolution. *Int. J. Biol. Macromol.* 242, 125014. https://doi./org/https://doi.org/10.1016/j.ijbiomac.2023.125014.

Raskin, D. M., Mishra, A., He, H., and Lundy, Z. (2020). Stringent response interacts with the ToxR regulon to regulate *Vibrio* *cholerae* virulence factor expression. *Arch. Microbiol*. 202, 1359–1368. https://doi./org/10.1007/s00203-020-01847-6.

Rocchio, S., Santorelli, D., Rinaldo, S., Franceschini, M., Malatesta, F., Imperi, F., Federici, L., Travaglini-Allocatelli, C., and Di Matteo, A. (2019). Structural and functional investigation of the small ribosomal subunit biogenesis gtpase a (rsga) from *Pseudomonas aeruginosa*. *Febs. J.* 286, 4245–4260. https://doi./org/10.1111/febs.14959.

Rocha, L. S., Silva, B. P. D., Correia, T. M. L., Silva, R. P. D., Meireles, D. A., Pereira, R., Netto, L. E. S., Meotti, F. C., and Queiroz, R. F. (2021). Peroxiredoxin ahpc1 protects *Pseudomonas aeruginosa* against the inflammatory oxidative burst and confers virulence. *Redox. Biol*. 46, 102075. https://doi./org/10.1016/j.redox.2021.102075.

Roy, R., You, R. I., Lin, M. D., and Lin, N. T. (2020). Mutation of the carboxy-terminal processing protease in *Acinetobacter baumannii* affects motility, leads to loss of membrane integrity, and reduces virulence. *Pathogens.* 9, 322. https://doi./org/10.3390/pathogens9050322.

Saha, M., Sarkar, S., Sarkar, B., Sharma, B. K., Bhattacharjee, S., and Tribedi, P. (2016). Microbial siderophores and their potential applications: A review. *Environ. Sci. Pollut. Res. Int*. 23, 3984–3999. https://doi./org/10.1007/s11356-015-4294-0.

Sahebi, M., Tarighi, S., and Taheri, P. (2022). The Arac-like transcriptional regulator YqhC is involved in pathogenicity of *Erwinia amylovora*. *J. Appl. Microbiol*. 132, 1319–1329. https://doi./org/10.1111/jam.15286.

Sakata, J., Yonekita, T., and Kawatsu, K. (2018). Development of a rapid immunochromatographic assay to detect contamination of raw oysters with enteropathogenic *Vibrio* *parahaemolyticus*. *Int. J. Food. Microbiol*. 264, 16–24. https://doi./org/10.1016/j.ijfoodmicro.2017.10.016.

Saleh, M. M., Sadeq, R. A., Latif, H. K. A., Abbas, H. A., and Askoura, M. (2019). Zinc oxide nanoparticles inhibits quorum sensing and virulence in *Pseudomonas aeruginosa*. *Afr. Health Sci*. 19, 2043–2055. https://doi./org/10.4314/ahs.v19i2.28.

Sánchez-Popoca, D., Serrano-Fujarte, I., Fernández-Mora, M., and Calva, E. (2022). The Leuo regulator and quiescence: About transcriptional roadblocks, multiple promoters, and crispr-cas. *Mol. Microbiol*. 118(5), 503–509. https://doi./org/10.1111/mmi.14990.

Sause, W. E., Balasubramanian, D., Irnov, I., Copin, R., Sullivan, M. J., Sommerfield, A., Chan, R., Dhabaria, A., Askenazi, M., Ueberheide, B., Shopsin, B., van Bakel, H., and Torres, V. J. (2019). The purine biosynthesis regulator purr moonlights as a virulence regulator in *Staphylococcus aureus*. *Proc. Natl. Acad. Sci. U.S.A.* 116, 13563–13572. https://doi./org/10.1073/pnas.1904280116.

Schachterle, J. K., and Sundin, G. W. (2019). The leucine-responsive regulatory protein Lrp participates in virulence regulation downstream of small RNA ArcZ in *Erwinia amylovora*. *Mbio*. 10, e00757-19. https://doi./org/10.1128/mBio.00757-19.

Shang, J., Wang, X., Zhang, M., Wang, R., Zhang, C., Huang, H., and Wang, S. (2021). Rid enhances the 6-hydroxypseudooxynicotine dehydrogenase reaction in nicotine degradation by *Agrobacterium tumefaciens* S33. *Appl. Environ. Microbiol*. 87, e02769-20. https://doi./org/10.1128/aem.02769-20.

Shukla, V., Asthana, S., Yadav, S., Rajput, V. S., and Tripathi, A. (2020). Emodin inhibited NADPH-quinone reductase competitively and induced cytotoxicity in rat primary hepatocytes. *Toxicon*. 188, 117–121. https://doi./org/10.1016/j.toxicon.2020.10.018.

Snead, K. J., Moore, L. L., and Bourne, C. R. (2022). Pard antitoxin hotspot alters a disorder-to-order transition upon binding to its cognate pare toxin, lessening its interaction affinity and increasing its protease degradation kinetics. *Biochemistry*. 61, 34–45. https://doi./org/10.1021/acs.biochem.1c00584.

Socea, J. N., Bowman, G. R., and Wing, H. J. (2021). VirB, a key transcriptional regulator of virulence plasmid genes in *Shigella flexneri*, forms DNA-binding site dependent foci in the bacterial cytoplasm. *J. Bacteriol*. 203, e00627-20. https://doi./org/10.1128/jb.00627-20.

Spínola-Amilibia, M., Araújo-Bazán, L., de la Gándara, Á., Berger, J. M., and Arias-Palomo, E. (2023). IS21 family transposase cleaved donor complex traps two right-handed superhelical crossings. *Nat. Commun*. 14(1), 2335. https://doi./org/10.1038/s41467-023-38071-x.

Stietz, M. S., Liang, X., Wong, M., Hersch, S., and Dong, T. G. (2019). Double tubular contractile structure of the type VI secretion system displays striking flexibility and elasticity. *J. Bacteriol*. 202, e00425-19. https://doi./org/10.1128/jb.00425-19.

Su, W., Kumar, V., Ding, Y., Ero, R., Serra, A., Lee, B. S. T., Wong, A. S. W., Shi, J., Sze, S. K., Yang, L., and Gao, Y. G. (2018). Ribosome protection by antibiotic resistance ATP-binding cassette protein. *Proc. Natl. Acad. Sci*. *U.S.A*. 115, 5157–5162. https://doi./org/10.1073/pnas.1803313115.

Taj, A., Jia, L., Sha, S., Wang, C., Ullah, H., Haris, M., Ma, X., and Ma, Y. (2022). Functional analysis and enzyme characterization of mannose-1-phosphate guanylyl transferase (manb) from *Mycobacterium tuberculosis*. *Res. Microbiol*. 173, 103884. https://doi./org/10.1016/j.resmic.2021.103884.

Tang, W., Liu, S., Yu, X., Yang, Y., Zhou, X., and Lu, Y. (2021). The basis of tolerance mechanism to metsulfuron-methyl in *Roegneria kamoji* (triticeae: Poaceae). *Plants*. 10, 1823. https://doi./org/10.3390/plants10091823.

Tao, F., Si, F. L., Hong, R., He, X., Li, X.Y., Qiao, L., He, Z. B., Yan, Z. T., He, S. L., and Chen, B. (2022). Glutathione s-transferase (GST) genes and their function associated with pyrethroid resistance in the malaria vector *Anopheles sinensis*. *Pest. Manag. Sci*. 78, 4127–4139. https://doi./org/10.1002/ps.7031.

Teper, D., Zhang, Y., and Wang, N. (2019). Tfmr, a novel TetR-family transcriptional regulator, modulates the virulence of *Xanthomonas citri* in response to fatty acids. *Mol. Plant Pathol*. 20, 701–715. https://doi./org/10.1111/mpp.12786.

Tian, M., Bao, Y., Li, P., Hu, H., Ding, C., Wang, S., Li, T., Qi, J., Wang, X., and Yu, S. (2018). The putative amino acid ABC transporter substrate-binding protein AapJ2 is necessary for *Brucella* virulence at the early stage of infection in a mouse model. *Vet. Res*. 49, 32. https://doi./org/10.1186/s13567-018-0527-9.

Tseng, C. C., Murni, L., Han, T. W., Arfiati, D., Shih, H. T., and Hu, S. Y. (2019). Molecular characterization and heterologous production of the bacteriocin peocin, a DNA starvation/stationary phase protection protein, from *Paenibacillus ehimensis* NPUST1. *Molecules*. 24, 2516. https://doi./org/10.3390/molecules24132516.

Tsevelkhoroloo, M., Xiaoqiang, L., Jin, X. M., Shin, J. H., Lee, C. R., Kang, Y., and Hong, S. K. (2022). Luxr-type SCO6993 negatively regulates antibiotic production at the transcriptional stage by binding to promoters of pathway-specific regulatory genes in *Streptomyces coelicolor*. *J. Microbiol. Biotechnol*. 32, 1134–1145. https://doi./org/10.4014/jmb.2205.07050.

Vimal, A., Siddiqui, M. H., Verma, A., and Kumar, A. (2021). Degradation product of curcumin restrain salmonella typhimurium virulent protein l-asparaginase. *J. Complement. Integr. Med*. 20, 413-424. https://doi./org/10.1515/jcim-2021-0172.

Wang, X., Chen, L., Liu, J., Sun, T., and Zhang, W. (2020). Light-driven biosynthesis of myo-inositol directly from CO(2) in *synechocystis* sp. PCC 6803. *Front. Microbiol*. 11, 566117. https://doi./org/10.3389/fmicb.2020.566117.

Wang, G., Fan, C., Wang, H., Jia, C., Li, X., Yang, J., Zhang, T., Gao, S., Min, X., and Huang, J. (2022a). Type VI secretion system-associated FHA domain protein TagH regulates the hemolytic activity and virulence of *Vibrio cholerae*. *Gut. Microbes*. 14, 2055440. https://doi./org/10.1080/19490976.2022.2055440.

Wang, J., Tian, Q., Cui, L., Cheng, J., Zhou, H., Peng, A., Qiu, G., and Shen, L. (2022b). Effect of extracellular proteins on Cd(II) adsorption in fungus and algae symbiotic system. *J. Environ. Manage*. 323, 116173. https://doi./org/10.1016/j.jenvman.2022.116173.

Westermann, A. J., Venturini, E., Sellin, M. E., Förstner, K. U., Hardt, W. D., and Vogel, J. (2019). The major RNA-binding protein proQ impacts virulence gene expression in *Salmonella enterica* serovar typhimurium. *Mbio*. 10, e02504-18. https://doi./org/10.1128/mBio.02504-18.

Xie, X., Hao, F., Chen, R., Wang, J., Wei, Y., Liu, J., Wang, H., Zhang, Z., Bai, Y., Shao, G., Xiong, Q., and Feng, Z. (2021). Nicotinamide adenine dinucleotide-dependent flavin oxidoreductase of *Mycoplasma hyopneumoniae* functions as a potential novel virulence factor and not only as a metabolic enzyme. *Front. Microbiol*. 12, 747421. https://doi./org/10.3389/fmicb.2021.747421.

Xu, Q., Chen, H., Sun, W., Zhang, Y., Zhu, D., Rai, K. R., Chen, J. L., and Chen, Y. (2021). sRNA23, a novel small RNA, regulates to the pathogenesis of *Streptococcus suis* serotype 2. *Virulence.* 12, 3045–3061. https://doi./org/10.1080/21505594.2021.2008177.

Xu, L., He, Q., Tang, Y., Wen, W., Chen, L., Li, Y., Yi, C., and Fu, B. (2022). Multi‑locus sequence and drug resistance analysis of salmonella infection in children with diarrhea in guangdong to identify the dominant st and cause of antibiotic‑resistance. *Exp. Ther. Med*. 24, 678. https://doi./org/10.3892/etm.2022.11614.

Xu, H., Huang, K., Lin, Y., Gong, H., Ma, X., and Zhang, D. (2023). Glycosyltransferase GLT8D1 and GLT8D2 serve as potential prognostic biomarkers correlated with tumor immunity in gastric cancer. *BMC Med. Genomics*. 16, 123. https://doi./org/10.1186/s12920-023-01559-y.

Xu, X., Li, H., Qi, X., Chen, Y., Qin, Y., Zheng, J., and Jiang, X. (2020a). Chea, cheb, cher, chev, and chey are involved in regulating the adhesion of *Vibrio harveyi.* *Front. Cell Infect. Microbiol*. 10, 591751. https://doi./org/10.3389/fcimb.2020.591751.

Xu, C., Soyfoo, D. M., Wu, Y., and Xu, S. (2020b). Virulence of helicobacter pylori outer membrane proteins: An updated review. *Eur. J. Clin. Microbiol. Infect. Dis*. 39, 1821–1830. https://doi./org/10.1007/s10096-020-03948-y.

Yahiro, K., Ogura, K., Terasaki, Y., Satoh, M., Miyagi, S., Terasaki, M., Yamasaki, E., and Moss, J. (2019). Cholix toxin, an eukaryotic elongation factor 2 ADP-ribosyltransferase, interacts with prohibitins and induces apoptosis with mitochondrial dysfunction in human hepatocytes. *Cell Microbiol*. 21, e13033. https://doi./org/10.1111/cmi.13033.

Yamasaki, S., Koga, N., Zwama, M., Sakurai, K., Nakashima, R., Yamaguchi, A., and Nishino, K. (2022). Spatial characteristics of the efflux pump MexB determine inhibitor binding. *Antimicrob. Agents Chemother*. 66, e0067222. https://doi./org/10.1128/aac.00672-22.

Yan, Y., Wang, H., Zhu, S., Wang, J., Liu, X., Lin, F., and Lu, J. (2019). The methylcitrate cycle is required for development and virulence in the rice blast fungus *Pyricularia oryzae*. *Mol. Plant. Microbe. Interact*. 32, 1148–1161. https://doi./org/10.1094/mpmi-10-18-0292-r.

Yu, P., Yang, L., Wang, J., Su, C., Qin, S., Zeng, C., and Chen, L. (2022). Genomic and transcriptomic analysis reveal multiple strategies for the cadmium tolerance in *Vibrio parahaemolyticus* N10-18 isolated from aquatic animal Ostrea gigas Thunberg. *Foods*. 11, 3777. https://doi./org/10.3390/foods11233777.

Yu, X., Ding, Z., Ji, Y., Zhao, J., Liu, X., Tian, J., Wu, N., and Fan, Y. (2020). An operon consisting of a P-type ATPase gene and a transcriptional regulator gene responsible for cadmium resistances in *Bacillus vietamensis* 151-6 and *Bacillus marisflavi* 151-25. *BMC Microbiol*. 20, 18. https://doi./org/10.1186/s12866-020-1705-2.

Zandi, T. A., Marshburn, R. L., Stateler, P. K., and Brammer Basta, L. A. (2019). Phylogenetic and biochemical analyses of mycobacterial l,d-transpeptidases reveal a distinct enzyme class that is preferentially acylated by meropenem. *ACS Infect. Dis*. 5, 2047–2054. https://doi./org/10.1021/acsinfecdis.9b00234.

Zhang, H., Zhao, C., Zhang, X., Li, J., Gong, P., Wang, X., Li, X., Wang, X., Zhang, X., Cheng, S., Yue, T., and Zhang, N. (2023). A potential role for Giardia chaperone protein GdDnaJ in regulating Giardia proliferation and Giardiavirus replication. *Parasit. Vectors*. 16, 168. https://doi./org/10.1186/s13071-023-05787-0.

Zhang, L., Zhao, G., Hu, X., Liu, J., Li, M., Batool, K., Chen, M., Wang, J., Xu, J., Huang, T., Pan, X., Xu, L., Yu, X. Q., and Guan, X. (2017). Cry11Aa interacts with the ATP-binding protein from culex quinquefasciatus to improve the toxicity. *J. Agric. Food Chem*. 65, 10884-10890. https://doi./org/10.1021/acs.jafc.7b04427.

Zhang, Y., Hong, Z., Zhou, L., Zhang, Z., Tang, T., Guo, E., Zheng, J., Wang, C., Dai, L., Si, T., and Wang, H. (2022). Biosynthesis of gut-microbiota-derived lantibiotics reveals a subgroup of S8 family proteases for class III leader removal. *Angew. Chem. Int. Ed. Engl*. 61, e202114414. https://doi./org/10.1002/anie.202114414.

Zhao, B. R., Wang, X. X., and Wang, X. W. (2022). Shoc2 recognizes bacterial flagellin and mediates antibacterial Erk/Stat signaling in an invertebrate. *PLOS Pathog*. 18, e1010253. https://doi./org/10.1371/journal.ppat.1010253.

Zheng, S., Nagao, J. I., Nishie, M., Zendo, T., and Sonomoto, K. (2018). ATPase activity regulation by leader peptide processing of ABC transporter maturation and secretion protein, NukT, for lantibiotic nukacin ISK-1. *Appl. Microbiol. Biotechnol*. 102, 763–772. https://doi./org/10.1007/s00253-017-8645-2.

Zhou, W., Fang, C., Zhang, L., Wang, Q., Li, D., and Zhu, D. (2020). Thioredoxin domain-containing protein 9 (TXNDC9) contributes to oxaliplatin resistance through regulation of autophagy-apoptosis in colorectal adenocarcinoma. *Biochem. Biophys. Res. Commun*. 524, 582–588. https://doi./org/10.1016/j.bbrc.2020.01.092.

Zhou, W., Shi, W., Xu, X. W., Li, Z. G., Yin, C. F., Peng, J. B., Pan, S., Chen, X. L., Zhao, W. S., Zhang, Y., Yang, J., and Peng, Y. L. (2018). Glutamate synthase MoGlt1-mediated glutamate homeostasis is important for autophagy, virulence and conidiation in the rice blast fungus. *Mol. Plant Pathol*. 19, 564–578. https://doi./org/10.1111/mpp.12541.

Ziegler, C. A., and Freddolino, P. L. (2021). The leucine-responsive regulatory proteins/feast-famine regulatory proteins: An ancient and complex class of transcriptional regulators in bacteria and archaea. *Crit. Rev. Biochem. Mol. Biol*. 56, 373–400. <https://doi./org/10.1080/10409238.2021.1925215.>
